# MVA.HIVconsvX vaccination–evoked T cell expansion inversely associates with age in people with HIV-1 on antiretroviral therapy

**DOI:** 10.1172/JCI193547

**Published:** 2026-05-05

**Authors:** Cynthia L. Gay, Yinyan Xu, Ann Marie K. Weideman, Fiona R. Shaw, JoAnn D. Kuruc, Shayla Z. Conrad, Sofia A. Mariano, Shahryar Samir, Sallay Kallon, Alexis T. Sponaugle, Joanna A. Warren, Genevieve T. Clutton, Maria Abad-Fernandez, Carolina Kapper, Alex B. Bradley, Caroline E. Baker, Susan M. Pedersen, Matthew J. Moeser, Lauren Burke, Edmund G.-T. Wee, Alison Crook, Gregory M. Laird, Joshua C. Cyktor, John W. Mellors, Shuntai Zhou, Lawrence Fox, Joseph J. Eron, David M. Margolis, Michael G. Hudgens, Tomáš Hanke, Nilu Goonetilleke

**Affiliations:** 1 Department of Medicine,; 2 University of North Carolina HIV Cure Center,; 3 Department of Microbiology and Immunology,; 4 Biostatistics Core, Center for AIDS Research, and; 5 Department of Biostatistics, University of North Carolina, Chapel Hill, North Carolina, USA.; 6 Global Statistical Sciences, Eli Lilly and Company, Indianapolis, Indiana, USA.; 7 Department of Radiology, University of North Carolina, Chapel Hill, North Carolina, USA.; 8 The Jenner Institute, Nuffield Department of Medicine, University of Oxford, Oxford, United Kingdom.; 9 Accelovir Diagnostics, Baltimore, Maryland, USA.; 10 Division of Infectious Disease, University of Pittsburgh, Pittsburgh, Pennsylvania, USA.; 11 HIV Research Branch, Division of AIDS, National Institute of Allergy and Infectious Diseases, NIH, Washington, DC, USA.

**Keywords:** AIDS/HIV, Aging, Immunology, AIDS vaccine, Clinical trials, T cells

## Abstract

**BACKGROUND:**

Approaches to achieving antiretroviral therapy–free (ART-free) remission from HIV-1 must consider that people over 50 years now comprise the majority of people with HIV (PWH) on ART in various regions, including the United States.

**METHODS:**

We report a double-blind, randomized trial in which PWH on ART, aged 21–60 years, received modified vaccinia Ankara–vectored (MVA-vectored) vaccines, MVA.tHIVconsv3 (M3) and MVA.tHIVconsv4 (M4), either alone or in combination (*n* = 7/group), or saline placebo (*n* = 3). M3 and M4 contain complementary HIVconsvX immunogens that each span the same regions in HIV-1 Gag and Pol but differ by approximately 8% at the amino acid level.

**RESULTS:**

M3, M4, and M3M4 regimens were well tolerated and all significantly increased both the frequency (peak median increase ~3-fold) and breadth of the HIVconsvX-specific T cell response while redirecting T cells to target conserved regions in HIV-1 for up to 10 weeks after vaccination. We also demonstrated that vaccination increased frequencies of T cells targeting participant autologous HIV-1 sequences. Vaccination mostly expanded preexisting HIV-1–specific T cells and did not impact CD4^+^ T cell activation, low-level viremia, or integrated HIV-1 provirus. Linear regression indicated that age was independently and negatively associated with the change in T cell frequency at 1, 2, and 10 weeks after vaccination (~1.41-fold decrease per 10 years older). After adjusting for age, years on ART was positively associated with HIVconsvX-specific T cell frequencies at 1 and 2 weeks following vaccination.

**CONCLUSION:**

In PWH receiving ART, MVA.HIVconsvX vaccines significantly increased T cells targeting conserved regions of HIV-1. Novel strategies may be required to enhance anti–HIV-1 immunity in older adults.

**TRIAL REGISTRATION:**

ClinicalTrials.gov NCT03844386

**FUNDING:**

NIH National Institute of Allergy and Infectious Diseases (NIAID) grants U01AI131310, HHSN272201100021I/HHSN27200037 (subcontract OX-14007.004.0037-212), UM1TR004406, P30AI050410, and P30CA016086; International AIDS Vaccine Initiative; European and Developing Countries Clinical Trials Partnership SRIA2015-1066; European Commission’s Horizon 2020 Research and Innovation Programme 681137.

## Abstract 2

This study demonstrates that new-generation vaccines can strongly increase immunity against HIV.

## Graphical abstract

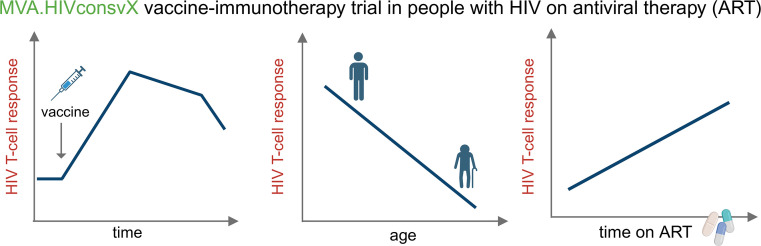

## Introduction

HIV-1 replication is successfully suppressed by antiretroviral therapy (ART). However, estimates suggest that natural clearance of cells harboring replication-competent virus in people with HIV-1 (PWH), collectively called the HIV-1 reservoir, takes more than 70 years (1). Multiple strategies are being evaluated to achieve ART-free HIV-1 suppression. A major focus is the development of immunotherapies and therapeutic vaccines designed to elicit effective CD8^+^ T cell immunity against HIV-1 to help clear HIV-1–infected cells and limit virus replication when off ART.

Multiple aspects of CD8^+^ T cell immunity have been associated with durable control of HIV-1. In PWH prior to ART initiation, increased breadth of HIV-1–specific CD8^+^ T cells targeting the structural protein Gag was correlated with lower viral load (2). HLA class I (HLA-I) alleles, which are highly polymorphic in humans, present virus-derived peptides to CD8^+^ T cells. Several HLA-I alleles, such as HLA-B*57:01 and HLA-B*57:03, are significantly enriched in PWH who exhibit sustained control of HIV-1 viremia off ART (3, 4). These protective alleles often present peptides derived from the highly conserved regions of HIV-1, where HIV-1 escape is associated with a fitness cost to the virus (5, 6). While protective HLA alleles are expressed by only a minority of individuals, vaccines that increase frequencies of CD8^+^ T cells targeting epitopes in these conserved regions increase selective pressure on HIV-1 (5, 7) and may improve virus control following ART treatment interruption (ATI).

We have designed and constructed modified vaccinia Ankara–vectored (MVA-vectored) vaccines expressing unique immunogens collectively known as HIVconsvX (8). The HIVconsvX immunogens consist of 6 functionally conserved HIV-1 regions: the entirety of p24 and a region in p15 of Gag, and 4 regions in Pol derived from protease, polymerase, and integrase. The conserved nature of these regions not only increases the likelihood of an epitope match between the immunogens and reservoir virus but also mitigates the effects of virus escape, including any preexisting escape in the HIV-1 reservoir (9). The HIVconsvX immunogens were designed in silico as mutually complementary bivalent mosaics. Vaccination with both mosaics covers a broader range of global HIV-1 variation than either one alone (8). In treatment-naive PWH from 4 continents, the presence of CD8^+^ T cells targeting the HIVconsvX regions was associated with lower HIV-1 virus load and greater immune preservation (higher CD4^+^ count) (10).

We previously combined recovery and full-length sequencing HIV-1 outgrowth viruses (OVs) with proteome mapping of HIV-1–specific T cell responses in a cohort of PWH on ART. On average, 39% of HIV-1–specific T cell epitopes in PWH on ART fell within the HIVconsvX regions (9). We also examined if HIV-1 variants in participant OVs were strongly or poorly recognized by circulating T cells, the latter indicating a virus variant that conferred T cell escape. Across the cohort, 32% of T cell epitopes harbored escape variants. T cell escape was not evenly distributed across the HIV-1 proteome, with only 15% of epitopes in HIVconsvX regions containing escape variants, 2.5-fold lower than non-HIVconsvX regions (9). Altogether, these studies suggest that HIVconsvX-targeting vaccines could improve circulating T cell immunity against the HIV-1 reservoir in PWH on ART.

Here, we present the results of the M&M Study, a double-blind, randomized phase I trial, involving PWH durably suppressed on ART. Participants received a single intramuscular vaccination of either MVA.tHIVconsv3 (M3), MVA.tHIVconsv4 (M4), or M3 and M4 together (M3M4). Vaccination was safe and well tolerated, and HIV-1–specific T cell responses in 85% of vaccinees were significantly (at least 2-fold) increased and redirected toward the HIVconsvX conserved regions. The M&M Study enrolled adults of diverse ages ranging from 21 to 60 years. We observed that participant age had a linear inverse correlation with the fold increase in vaccine-specific T cell response. These results have broader implications for the design of T cell vaccines and immunotherapies aimed at older individuals, who now comprise the majority of PWH in various regions, including the United States (11).

## Results

### Study design and cohort.

Twenty-nine participants were enrolled, with 25 completing the study; this included 1 participant who received undisclosed vaccination within 14 days of enrollment constituting an enrollment violation (Supplemental Figure 1; supplemental material available online with this article; https://doi.org/10.1172/JCI193547DS1). Three participants withdrew or were discontinued prior to vaccination due to scheduling conflicts, and 1 participant withdrew after vaccination at day 7. Participant clinical and demographic data are detailed in Table 1 and Supplemental Table 1. Participants were mostly male (87.5%); over half (54.2%) were Black or African American and 13.8% were Hispanic, with a median CD4^+^ count of 773.5 cells/mm^3^ (IQR 307.5). Despite randomization and an overall broad participant age, ranging from 21 to 60 years of age (median age 41), participants in the M3M4 group (median age 31) were, on average, a decade younger than M3-only (median age 43) and M4-only vaccinees (median age 46). The M3M4 group also contained more participants (*n* = 3) who started ART during acute HIV infection, with no participants in the M3 group and 1 in the M4 group who initiated ART during acute HIV infection (Table 1 and Supplemental Table 1). Four vaccinees (1 each in M3 and M4 and 2 in M3M4) expressed protective HLA alleles (Table 1 and Supplemental Table 1). Overall, participants had documented ART-mediated virus suppression from 2.37 to 18.12 years at enrollment (Table 1 and Supplemental Table 1). Over the course of this study, HIV-1 RNA remained <40 copies/mL plasma in all participants except in participant 1264 (M4 arm), who exhibited viral blips of 150 and 53 copies/mL at days 70 and 86, respectively, that were not judged related to study intervention (discussed further below).

### Safety and tolerability.

Consistent with the broad literature summarizing the MVA vector clinical safety profiles (12), vaccination across all 3 vaccine arms was safe and well tolerated. Most adverse events (AEs) were grade 1 (mild, 89%) or grade 2 (moderate, 6%) and resolved within 48 hours (Table 2 and Supplemental Table 2). Grade 3 (severe) AEs, possibly related to study treatment and consistent with reactogenicity, occurred in 4 participants and resolved within 24 to 48 hours. No AE of special interest or serious AEs occurred.

### Circulating HIVconsvX-specific T cell frequencies were significantly increased following the M3/M4/M3M4 vaccination.

**Figure 1:**
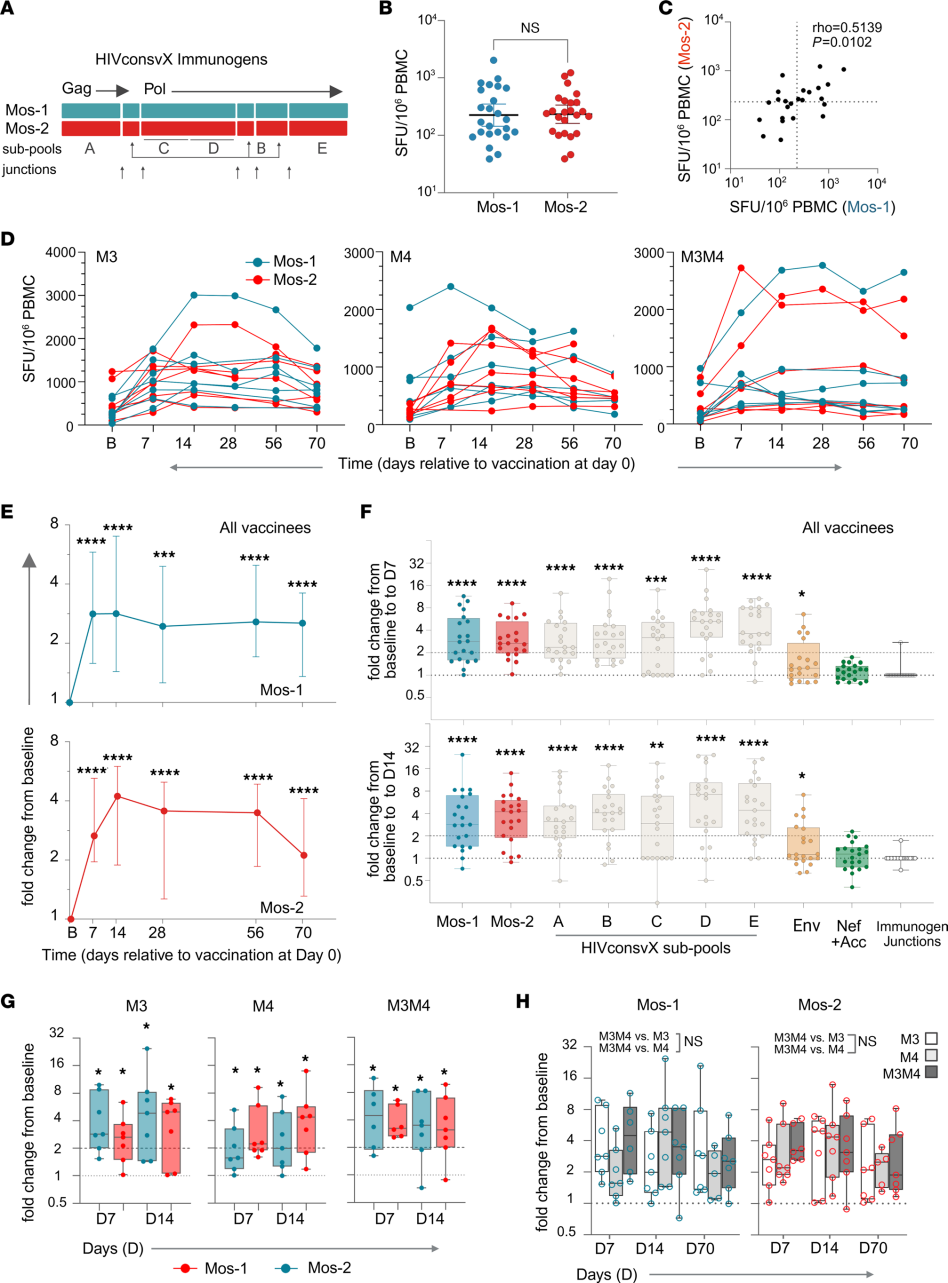
M3, M4, and M3M4 vaccinations increase the frequency of HIV-1–specific T cells in PWH on ART. (**A**) T cell frequencies were quantified by ex vivo IFN-γ ELISpot against peptides spanning the Mos-1 and Mos-2 immunogens and subpools A–E. (**B**) Baseline Mos-1– and Mos-2–specific T cell frequencies; all participants (*n* = 24) mean (±SD). Baseline was defined as the average of 1–3 (median = 3) prevaccination visits, mostly from day –0 to day –85. (**C**) Spearman’s correlation between Mos-1– and Mos-2–specific T cell frequencies measured at baseline. (**D**) Mos-1– and Mos-2–specific T cell frequencies (mean) for individual vaccinees in M3, M4, and M3M4 groups (*n* = 7/group) from baseline to 70 days following vaccination at day 0. (**E**) Fold change in Mos-1– and Mos-2–specific T cell frequencies (median, IQR) in all vaccinees (*n* = 21) from baseline to day 70. (**F**) Box-and-whisker plots of day 7 and 14 postvaccination T cell responses in vaccinees to Mos-1, Mos-2, subpools A–E, HIV-1 Env, Nef + Acc (Rev, Tat, Vpr, Vpu, Vif), and peptides spanning junctions within immunogens. (**G**) Fold change from baseline, within vaccine group. (**H**) Fold change from baseline, between vaccine groups. The box-and-whisker plots depict the minimum and maximum values (whiskers), the upper and lower quartiles, and the median. (**B** and **H**) Wilcoxon’s rank-sum test for between-group testing; (**E**–**G**) 2-sided exact Wilcoxon’s signed-rank test for within-group testing. In **F**–**H**, dotted lines highlight 2-fold or no change (1-fold) from baseline. NS, nonsignificant or *P* > 0.05, *P < 0.05, **P < 0.005, ***P < 0.0005, ****P < 0.00005.

T cell responses to the HIVconsvX peptide pools (Figure 1A), Mos-1 (mosaic 1 as in M3) and Mos-2 (mosaic 2 as in M4), were measured from before to 10 weeks after vaccination using an ex vivo IFN-γ enzyme-linked immunospot (ELISpot) assay. All participants harbored T cell responses to both Mos-1 and Mos-2 peptides prior to vaccination (Figure 1B). Consistent with the high (92%) amino acid identity between the immunogens, Mos-1– and Mos-2–specific T cell frequencies prior to vaccination were similar and significantly correlated (Figure 1, B and C).

Kinetics of Mos-1– and Mos-2–specific T cell frequencies following vaccination for participants are shown in Figure 1D. Baseline T cell frequencies for participants were calculated as the average of 1–3 (median = 3) prevaccination visits, mostly between day –85 and day 0 (day of vaccination). Across participants, T cell response to M3/M4/M3M4 vaccinations mostly peaked between 7 and 14 days following vaccination (Figure 1, D and E). On day 7, the median fold increase in T cell frequency from baseline was 2.81 (IQR 3.58) for Mos-1 and 2.66 (IQR 1.85) for Mos-2. On day 14, the median fold increase in T cell frequency was 2.83 (IQR 3.99) for Mos-1 and 4.23 (IQR 4.01) for Mos-2 (Figure 1, D and E). Overall, 18/21 (86%) vaccinees produced a 2-fold or greater increase in T cell frequencies to 1 or both HIVconsvX immunogens (Supplemental Table 3). Median vaccine-specific T cell frequencies remained elevated (Mos-1, median 2.52, IQR 1.72; Mos-2, median 1.11, IQR 2.12) through the last measurement at day 70 (Figure 1, D and E, and Supplemental Table 3). T cells specific for all 5 Mos-1 and Mos-2 subpools (Figure 1A) were also significantly increased following vaccination (Figure 1F). By contrast, T cell frequencies to Mos-1 and Mos-2 measured in the placebo recipients exhibited < 2-fold change across all postvaccination time points (Supplemental Figure 2, A–C, and Supplemental Table 3).

Vaccine-induced increases in Mos-1– and Mos-2–specific T cells were detected in each vaccine group (all comparisons, *n* = 7/group, *P* ≤ 0.04; Figure 1G). However, no difference was observed to vaccination between vaccine groups, suggesting that vaccine regimens resulted in comparable increases in HIVconsvX-specific T cell frequencies (Figure 1H).

We examined the induction of T cells specific for irrelevant junctional neoepitopes within the HIVconsv3 and HIVconsv4 immunogens indicated in Figure 1A. Only 1 participant developed a T cell response to peptides spanning regional junctions, suggesting that these non–HIV-1 sequences are poorly immunogenic (Figure 1F). At all visits, peptides derived from HIV-1 regions outside of the vaccine immunogens were also tested. Vaccination induced no postvaccination change in T cells targeting HIV-1 Nef and accessory proteins (6 HIV-1 proteins tested together in a single peptide pool), but a transient increase in Env-specific T cells was found (*n* = 21; day 7, *P* = 0.009; day 14, *P* = 0.023; Figure 1F) that returned to baseline by day 70 (*P* = 0.144; data not shown).

In summary, M3/M4/M3M4 significantly increased HIVconsvX-specific T cell responses in study participants for at least 10 weeks after vaccination.

### HIVconsvX-specific T cell breadth significantly increased following the M3/M4/M3M4 vaccinations.

**Figure 2:**
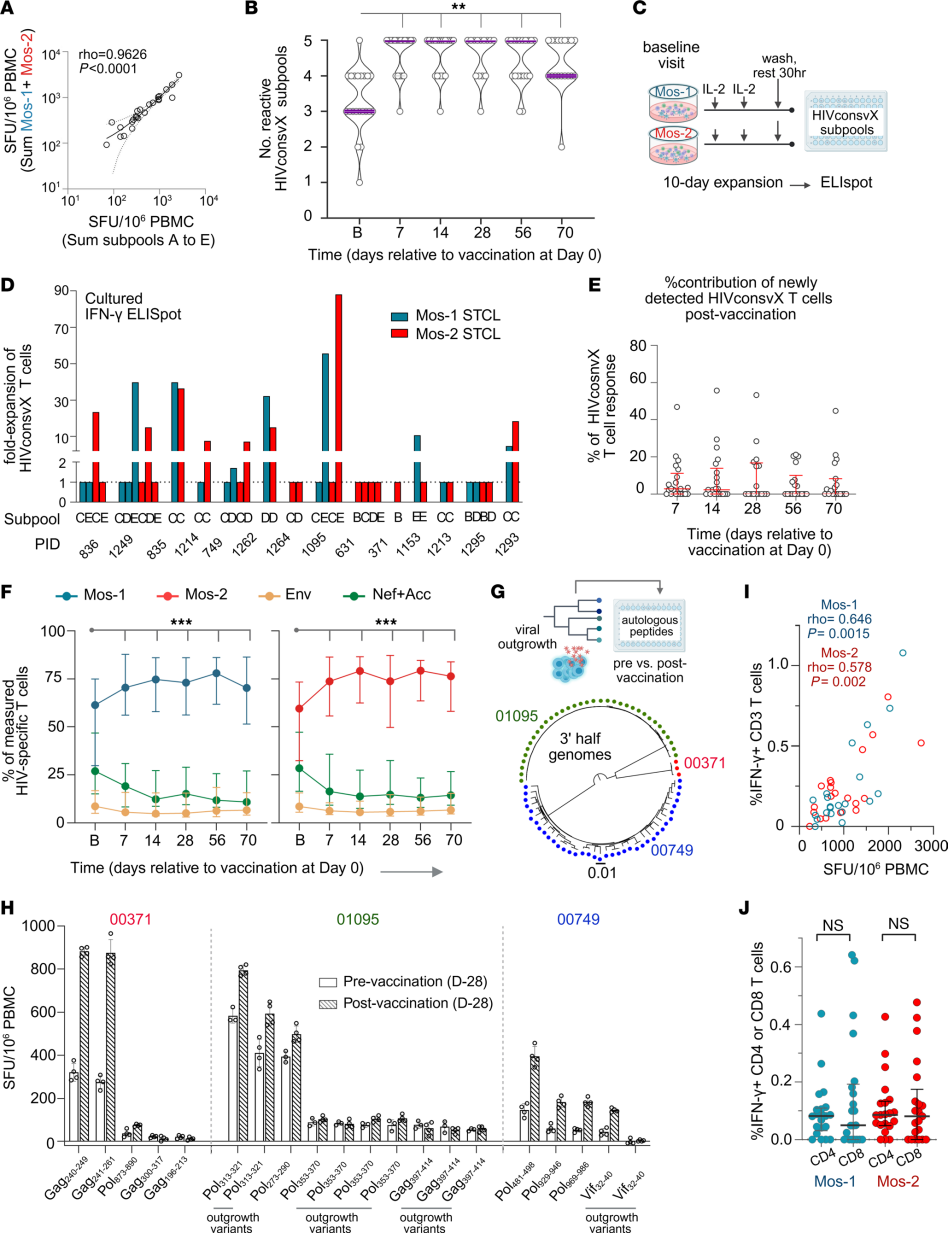
M3/M4/M3M4 vaccination increased the breadth and immunodominance of Mos-1– and Mos-2–specific CD4^+^ and CD8^+^ T cells. T cell breadth and immunodominance were measured by ex vivo IFN-γ ELISpot. (**A**) Spearman’s correlation between sum of Mos-1– + Mos-2–specific T cells and the sum of T cells targeting HIVconsvX subpools A–E at baseline (*n* = 24); solid line = slope, dotted lines = 95% CI. (**B**) T cell breadth (median, IQR), all vaccinees (*n* = 21). (**C**) Schema of cultured ELISpot. (**D**) Fold expansion of T cells in Mos-1– (blue) or Mos-2–stimulated (red) STCLs to HIVconsvX subpools, relative to baseline ex vivo frequencies. (**E**) Percent contribution (median, IQR) of newly detected subpools to total HIVconsvX T cell frequencies after excluding subpools detected in cultured ELISpot. (**F**) Percent contribution (median, IQR) of T cells against either Mos-1 or Mos-2 to the total measured HIV-specific T cell responses in vaccinees (*n* = 21) at indicated visits. Acc, peptides spanning Rev, Tat, Vif, Vpu, and Vpr. (**G**) Approach and 3′ half genome phylogenetic trees. (**H**) Baseline and postvaccination T cell frequency (mean ± SEM) in participants 371, 1095, and 749 against mapped epitopes occurring in participants’ OVs. Peptide positions are indicated by HXB2 location. Peptide sequences are provided in Supplemental Table 4. (**I**) Spearman’s correlation between Mos-1 (blue) and Mos-2 (red) T cell frequency measured in ex vivo ELISpot and percent IFN-γ^+^ CD3 T cells by ICS measured at corresponding postvaccination visits (days 7–56) (*n* = 21). (**J**) Percent frequency (median, IQR) of Mos-1– and Mos-2–specific CD4^+^ and CD8^+^ T cells measured by ICS at day 7–56 after vaccination in all vaccinees (*n* = 21). (**B** and **F**) Wilcoxon’s signed-rank test; (**J**) Wilcoxon’s rank-sum test. NS, nonsignificant or *P* > 0.05, **P < 0.005, ***P < 0.0005. Baseline was defined as the average of 1–3 (median = 3) prevaccination visits, mostly from day –0 to day –85.

We defined ex vivo T cell breadth as the number of HIVconsvX-specific subpools detected in ELISpot (Figure 1A). Notably, T cell responses to Mos-1 and Mos-2 peptide pools were highly correlated with the sum of the subpools reflecting a maintenance of assay sensitivity across different pool sizes (*n* = 24, ρ = 0.9626, *P* < 0.0001; Figure 2A).

Vaccination increased the number of reactive subpools in vaccinees, overall increasing breadth from 3/5 subpools at baseline to 5/5 subpools after vaccination (*n* = 21, *P* < 0.005 at days 7, 14, 28, and 70; Figure 2B). Increases in breadth were also observed in each vaccine group, though most differences did not reach statistical significance, likely due to small group sizes (*n* = 7/group; Supplemental Figure 2D). Change in T cell breadth following vaccination was not separable among M3, M4, and M3M4 groups (data not shown).

While vaccination increased the average number of HIVconsvX subpools detected across participants, the magnitude of newly detected T cell responses measured by ex vivo ELISpot was relatively low and contributed a median of only 5% of the total ex vivo HIVconsvX-specific T cell response in vaccinees across postvaccination visits (data not shown).

We examined if these newly ex vivo detected T cell responses reflected de novo induction following vaccination or an increase of preexisting but low-frequency HIVconsvX-specific T cells. To examine this, we generated short-term T cell lines (STCLs) from a prevaccination visit in the 18 vaccinees in whom we detected putative new T cell responses in response to vaccination (Figure 2C). STCLs reliably expand both CD4^+^ and CD8^+^ T cells, including T cells targeting HIV-1, in culture (13). Two STCLs were generated in each participant: 1 stimulated with Mos-1 peptides and the other stimulated with Mos-2 peptides. After 10 days of culture, STCLs were stimulated overnight with HIVconsvX subpools (or mock controls), and T cell frequencies were measured by ELISpot (Figure 2D). All STCLs were tested against subpool A (conserved regions in Gag). All 18 participants had an ex vivo T cell response against this subpool (Supplemental Table 4). Detection of subpool A–specific T cells therefore served as a positive control for HIV-specific T cell expansion. We observed expansion of subpool A–specific T cells in 14/18 participants (Supplemental Table 4). The lack of expansion in the 4 participants, none of whom initiated ART during acute infection (Supplemental Table 1), may reflect proliferation defects in T cells associated with chronic HIV-1 infection prior to ART initiation (14). In the 14 participants with subpool A–specific T cell expansion, we detected responses to putative new subpools in 9 participants (Figure 2D), indicating that these individuals had low-frequency memory HIVconsvX-specific T cells against these subpools before vaccination.

When these preexisting HIVconsvX-specific T cells were excluded, the percent median contribution of new T cell responses to the total HIVconsvX response after vaccination lowered to approximately 2% (IQR 14) across postvaccination visits (Figure 2E). We conclude that MVA.HIVconsvX vaccines given alone or in combination overwhelmingly (~98%) expanded memory HIV-1–specific T cells.

### M3/M4/M3M4 vaccination shifted T cell immunodominance to conserved regions of HIV-1.

We summed T cell frequencies to either Mos-1 or Mos-2 (each spanning ~50% HIV-1 Gag and Pol proteins) with Env, Nef, and the accessory proteins Rev, Tat, Vif, Vpu, and Vpr. This calculation provided a near-total estimate of the proteome-wide HIV-1–specific T cell response in each participant. Prior to vaccination, the median contribution of T cells targeting the HIVconsvX regions was approximately 60% (mean ~50%) of the total measured HIV-1–specific T cell response (Figure 2F). This is consistent with our previous studies of PWH on ART that reported that T cell responses to these Gag and Pol regions contributed to half of the overall T cell magnitude to HIV-1 (9). Despite this high baseline, vaccination successfully increased the median proportion of T cells targeting the HIVconsvX regions by approximately 20% across vaccinees at days 7 and 14 (*n* = 21, *P* < 0.0005, days 7 and 14; Figure 2F). This effect was maintained over 10 weeks (Figure 2F). Increases in the immunodominance of T cells targeting Mos-1 and Mos-2 were also observed within each vaccine group at days 7 and 14 and some later visits (Supplemental Figure 2, E–J). No significant differences in change in Mos-1 or Mos-2 immunodominance were observed between groups (data not shown). In summary, all vaccinations successfully, and equally, shifted the T cell immunodominance, refocusing them on highly conserved HIV-1 regions.

### Vaccination increased detection of autologous reservoir virus within participants.

Our results thus far showed that M3, M4, and M3M4 vaccination was immunogenic, increasing T cell frequencies in most recipients against peptides matched to vaccine immunogens. Next, we combined sequencing of autologous HIV-1 OVs and epitope mapping to examine if vaccination also increased T cell targeting of epitopes in the HIV-1 reservoir of participants. We previously recovered and sequenced individual autologous, replication-competent OVs in 2 participants, 749 (M4) and 1095 (M4) (9). Both 749 and 1095 initiated ART in chronic infection and have complex HIV-1 reservoirs (Figure 2G and Supplemental Figure 3A) with within group pairwise distance for 3′ halves of 0.00105 and 0.01400, respectively (Supplemental Figure 3B) (9). We also recovered and sequenced the OVs from a third vaccinee, 371 (M3M4). Participant 371 had initiated ART in acute HIV infection (Fiebig stage III) (15), and consistent with acute staging, sequencing confirmed individual OVs to be genetically near-identical with within 3′ pairwise distance of 0.00099 (Figure 2G and Supplemental Figure 3, A and B) (16, 17).

In all 3 participants, we measured T cell responses to individual epitopes (testing the optimal CD8^+^ T cell epitope, if identified) matched to the OV sequences prior to and following vaccination. Variants detected in OVs were also tested (Figure 2G and Supplemental Table 5).

Participant 749 responded strongly to M4 vaccination, producing a peak 5.8- and 5.2-fold increase in Mos-1– and Mos-2–specific T cell frequencies at day 7 following vaccination (Supplemental Table 3). We detected 4 reactive T cell epitopes in 749’s HIV reservoir (epitope sequences detailed in Supplemental Table 5). Three epitopes occurred in HIVconsvX regions, and 1 epitope was outside of HIVconsvX, in Vif. The 3 HIVconsvX epitopes appeared in all 15 sequenced autologous OVs, consistent with these regions being highly conserved. The non-HIVconsvX Vif epitope contained a minor variant (2/15 sequences) that was not recognized, illustrating how viral variants can lead to T cell escape in the HIV-1 reservoir. Consistent with the significant increase in T cell frequencies to Mos-1 and Mos-2 peptide pools observed following vaccination, T cell frequencies measured against mapped epitopes within the reservoir increased approximately 3-fold 4 weeks after M4 vaccination (Figure 2H). An increase in the reactive Vif epitope was also observed.

Participant 1095 was 1 of just 3 participants who did not respond (<2-fold change in T cell frequency) to MVA.HIVconsvX vaccination, producing a small 1.6-fold increase in Mos-2–specific T cells 7 days following M4 vaccination (Supplemental Table 3). Four reactive T cell epitopes were detected in OVs prior to vaccination. All reactive epitopes occurred in HIVconsvX regions. Virus variants were detected in 3/4 of these regions, including the immunodominant T cell epitope Pol303-321, for which we had confirmed the optimal 9-mer length. Consistent with the weak global response to M4 vaccination, T cell frequencies against both Pol303-321 variants (T/I at position 320) and the next most immunogenic epitope Pol273-290 (no variants were detected) increased, but the fold change measured at day 28 was small, less than 1.5-fold. T cell frequencies against 2 subdominant epitopes Gag397-414 and Pol353-370, both of which contained multiple variants, were not increased by vaccination (Figure 2H).

Participant 371 responded strongly to M3M4 vaccination, producing a 7.6- and 3.4-fold increase in Mos-1– and Mos-2–specific T cells 7 days following vaccination (Supplemental Table 3). We detected 5 reactive T cell epitopes in OVs, all occurring in HIVconsvX regions. We identified the optimal immunodominant epitope as Gag240-249 (TW10), restricted by either HLA-B*57:01 or HLA-B*58:01; both are protective HLA alleles, and both were expressed by PID371 (Supplemental Table 4). Consistent with the strong global T cell response to M3M4 vaccination, T cell frequencies measured against TW10 and the next most immunogenic epitope, Gag244-261, were increased by almost 3-fold at 4 weeks following vaccination (Figure 2H).

In summary, we show that HIVconsvX immunogens targeted immunodominant T cell epitopes in OVs of all 3 participants examined, consistent with our previous larger study (9). We also confirmed that MVA.HIVconsvX vaccine responders also increased frequencies of T cells targeting participant’s HIV reservoirs.

### Postvaccination HIVconsvX-specific T cells comprised both CD4^+^ and CD8^+^ T cells.

The CD8^+^ or CD4^+^ profile of HIVconsvX-specific T cells in all vaccinees was measured following vaccination by intracellular cytokine staining (ICS) for IFN-γ and the cytolytic granule marker CD107a. To maximize assay sensitivity, ICS was performed at or near the peak HIVconsvX-specific T cell response (7–56 days after vaccination, median 14 days), as measured by ELISpot for each participant (Supplemental Table 6). Notably, Mos-1 and Mos-2 IFN-γ^+^ T cells measured by ICS and ELISpot correlated significantly, cross-validating both methods (Figure 2I). HIVconsvX-specific T cells were evenly distributed between CD4^+^ and CD8^+^ T cells (Figure 2J and Supplemental Table 6). Consistent with the established cytolytic profile of CD8^+^ T cells, IFN-γ^+^ HIVconsvX-specific T cells mostly coexpressed CD107a, while IFN-γ^+^CD107a^+^ coexpression was lower in CD4^+^ T cells (Supplemental Table 6). In 17/21 vaccinees, both CD4^+^ and CD8^+^ HIVconsvX-specific T cells, either single IFN-γ^+^ or IFN-γ^+^CD107a^+^ were detected (Supplemental Table 6). Among the remaining 4 vaccinees, CD8^+^ HIVconsvX T cells were detected in only 1 individual, while CD4^+^ HIVconsvX T cells were only detected in only 2 vaccinees, and in 1 vaccinee no HIVconsvX T cells were detected. This last vaccinee, 01153, had ex vivo detectable HIVconsvX-specific T cells in the ELISpot assay that were low frequency but likely below the level of sensitivity of our ICS (Supplemental Table 6). In summary, immunization with MVA.HIVconsvX vaccines given alone or in combination increased circulating frequencies of both HIV-specific CD4^+^ and CD8^+^ T cells.

### Age was inversely associated with change in T cell frequency following M3/M4/M3M4 vaccination.

**Figure 3:**
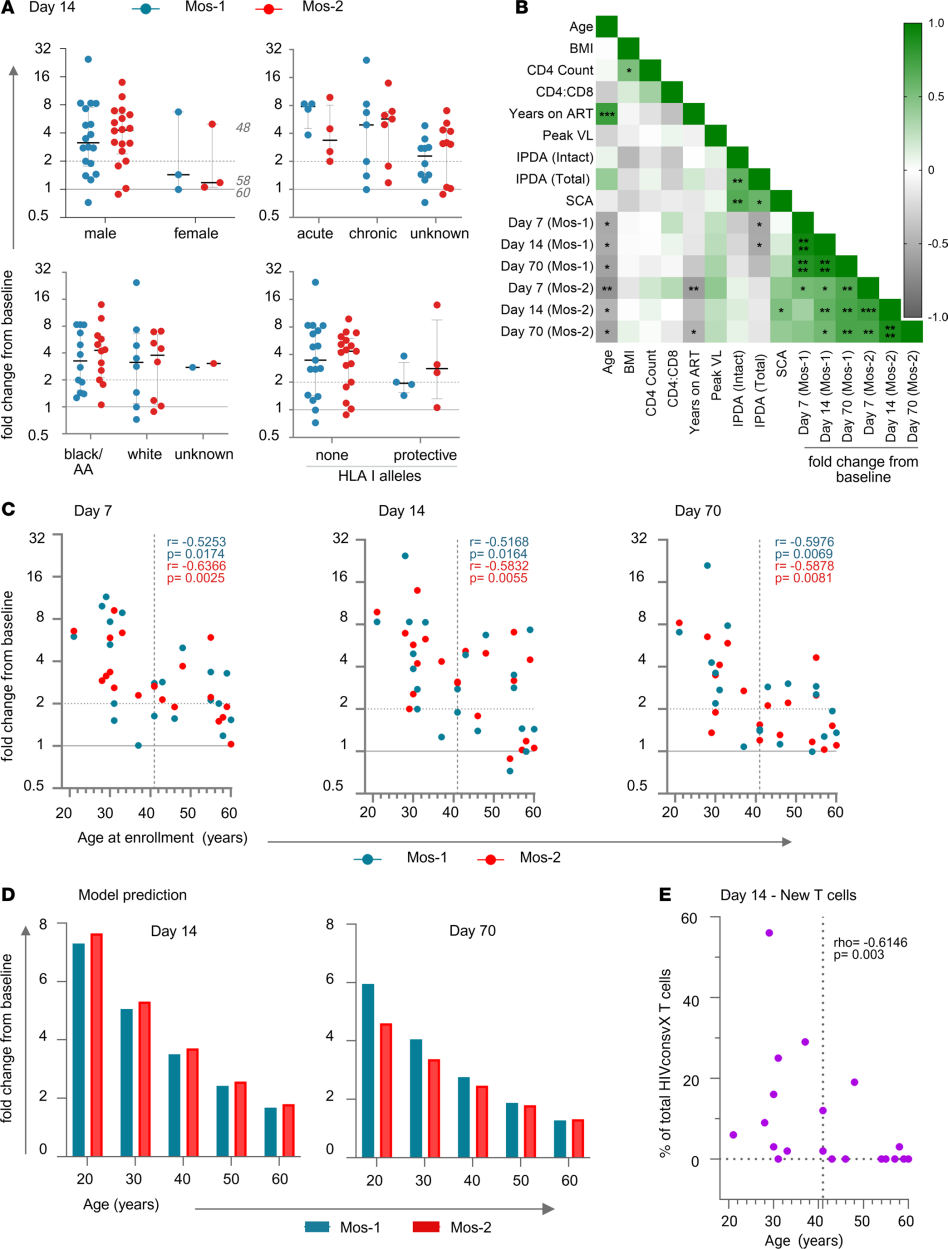
Age is linearly and inversely correlated with T cell response to vaccination. (**A**) Comparisons of the categorical variables sex at birth (top left), race (bottom left), HIV-1 staging at ART initiation (top right), and protective HLA I alleles (bottom right) with fold change in T cell response (median, IQR) to Mos-1 and Mos-2 at day 14, measured by ex vivo IFN-γ ELISpot (*n* = 21). Ages of the 3 female participants are displayed in gray beside their corresponding fold change in Mos-2 T cell frequency. (**B**) Spearman’s rank-order correlations between log_2_FC in T cell response to Mos-1 and Mos-2 (*n* = 21), baseline virologic measurements (SCA, *n* = 20; IPDA, *n* = 17), and vaccinee demographic and clinical characteristics (*n* = 21). VL, viral load. *P < 0.05, **P < 0.005, ***P < 0.0005. (**C**) Pearson’s correlation between age at enrollment and fold change in Mos-1– and Mos-2–specific T cell frequencies from baseline to days 7, 14, and 70 after vaccination. Horizontal dotted lines correspond to 2-fold change relative to participant baseline. Vertical line = median age of 41 years. (**D**) Linear regression slope estimates from day 14 and 70 data, detailed in Table 3, were used to estimate the impact of age on Mos-1– and Mos-2–specific T cell response to MVA.HIVconsvX vaccination. See also Supplemental Table 7. (**E**) Spearman’s rank correlation age at enrollment and percentage of new T cell responses [log_2_(% + 0.1)] against both Mos-1 and Mos-2 to vaccination at day 14 after vaccination. Baseline was defined as the average of 1–3 (median = 3) prevaccination visits, mostly from day –0 to day –85. AA, African American; acute, participant in acute HIV-1 infection at ART initiation; chronic, participant in chronic HIV infection at ART initiation; unknown, HIV status unknown at ART initiation.

Across participants, we observed a wide range in fold change of 0–24 in T cell frequency to the HIVconsvX regions following vaccination (Figure 1, D–F). While our group sizes were too low for statistical analysis, individual graphing of the fold change in HIVconsvX-specific T cells following vaccination suggests that the vaccination response was not strongly skewed by categorical variables of sex, ethnicity, HIV staging at time of ART initiation, or the expression of protective HLA alleles (Figure 3A).

We next correlated other participant demographics (continuous variables) with T cell response to MVA.HIVconsvX vaccination using nonparametric, rank-order correlation (Figure 3B and Supplemental Figure 4A). While these analyses focused on T cell response to Mos-1 and Mos-2, we note that fold change in T cell frequency to Mos-1, Mos-2, and subpools and the percentage of new T cells were all strongly associated (Supplemental Figure 4B). Both age at enrollment and time on ART were associated with fold change of T cell frequency to Mos-1 and Mos-2 (Figure 3B). Age and time on ART also directly correlated (ρ = 0.77, *P* < 0.005 Spearman’s rank; Supplemental Figure 4C).

Following checks for linearity, Pearson’s correlations of T cell response to vaccination and participant age showed significant inverse associations at 7, 14, and 70 days after vaccination (Figure 3C and Table 3). Linear regression analysis at multiple postvaccination time points predicted an approximately 0.5 decrease in log_2_ fold change or approximately 1.41-fold decrease in T cell frequency to both HIVconsvX immunogens with every 10 years of age (Table 3). Another way to describe this association is that whether PWH on ART is 20, 30, or 40 years of age, their T cell response to MVA.HIVconsvX vaccination is predicted to approximately halve over the subsequent 20 years (Figure 3D and Supplemental Table 7).

As noted above, most older participants had been on ART for a longer period than younger participants (Supplemental Figure 4C). However, when we adjusted for age, analysis of T cell frequencies to Mos-1 showed that a longer time on ART independently and positively associated with T cell response to vaccination (Table 3). This suggests that if age at enrollment was held constant, each additional year of ART would increase the fold change in Mos-1–specific T cells following vaccination. For T cell responses to Mos-2, years on ART did not independently predict the T cell frequencies (Table 3), possibly due to the high correlation between age and years on ART (Supplemental Figure 4C).

CD4^+^ T cell reconstitution and BMI are more broadly associated with immune health in PWH. We did not observe associations between CD4^+^ nadir, CD4^+^/CD8^+^ ratio, or BMI with fold change of the T cell response to vaccination or age or years on ART (Figure 3B and Supplemental Figure 4, D–G).

### New T cell responses detected following M3/M4/M3M4 vaccination also inversely associated with age.

De novo T cell response to vaccination and infection diminishes with age, attributed in part to thymic involution reducing naive T cell output (18). Our combined ex vivo and cultured ELISpot approaches showed that while MVA.HIVconsvX vaccination mostly expanded preexisting HIV-specific T cells within participants, de novo induction of HIV-1–specific T cells was indicated in some participants (Figure 2, A–E). We correlated participant age against percentage of newly detected HIVconsvX subpools in all vaccinees at day 14 after vaccination (Figure 3E). We observed a significant, inverse correlation (*n* = 21, ρ = –0.6146, *P* = 0.003, Spearman’s rank), suggesting that in older PWH on ART, MVA.HIVconsvX vaccination is less effective at expanding T cell breadth.

### M3/M4/M3M4 vaccinations were associated with a small increase in intact integrated provirus not reflected by total integrated provirus or by changes in low-level viremia.

**Figure 4:**
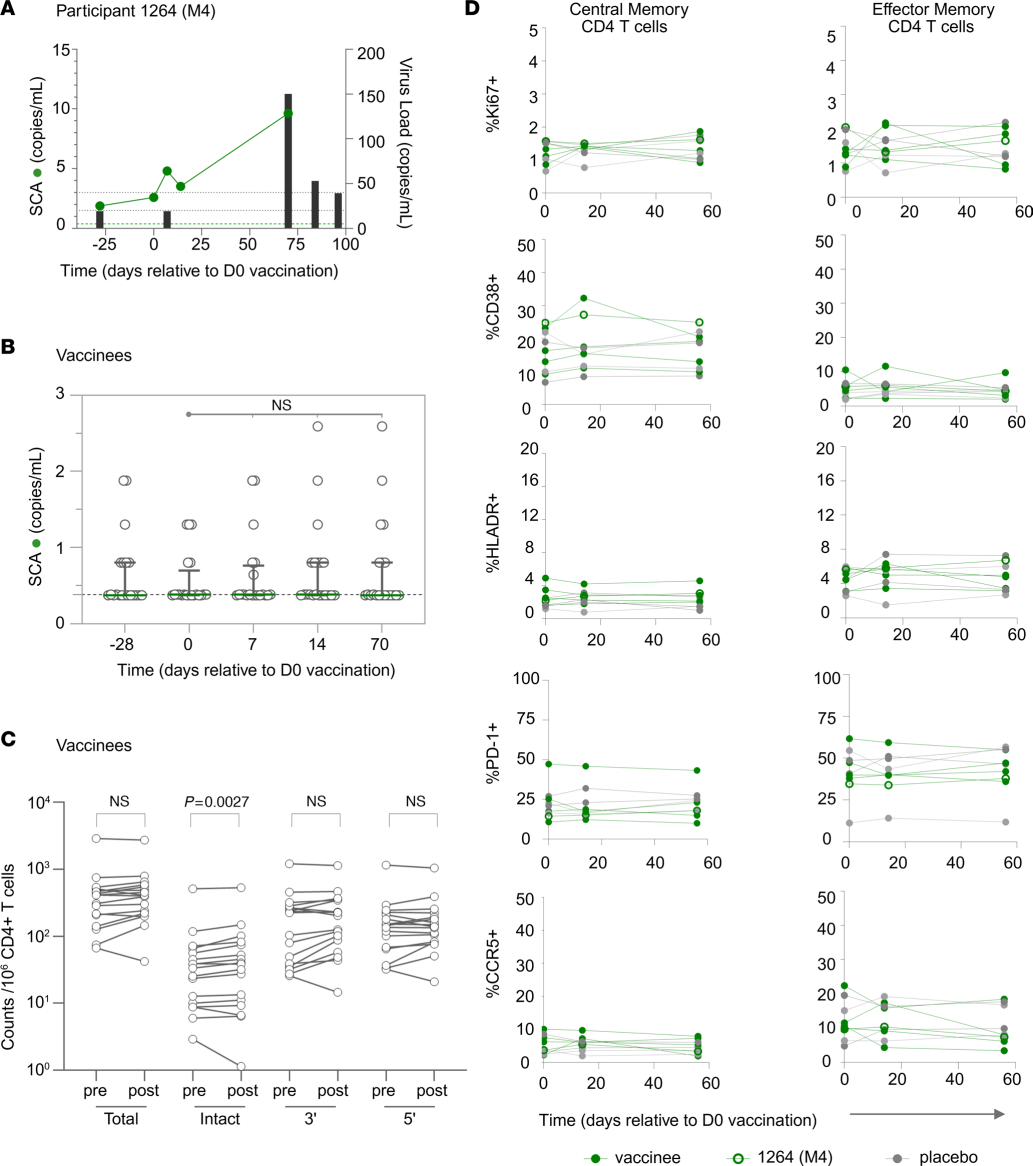
Vaccination levels did not impact persistent HIV-1 or CD4^+^ T cell activation. (**A**) Plasma virus load and low-level plasm viremia measured by SCA in participant 1264. Green dotted line indicates SCA threshold (0.38 copies/mL), and black dotted lines indicate 20 and 40 copies/mL in HIV RNA testing. (**B**) SCA (median, IQR) before (–28 days, D0) and after vaccination (*n* = 20 vaccinees, excluding 1264). Dotted line indicates SCA threshold (0.38 copies/mL). (**C**) Levels of integrated, total, and defective HIV-1 provirus in vaccinees (*n* = 17) measured at a single baseline visit (pre, –56 to –4 days before vaccination; median, –7 days) and a single postvaccination visit (post, 16–64 days after vaccination; median 28 days), measured by IPDAs. (**D**) Levels of Ki67, CD38, HLA-DR, PD-1, and CCR5 in central (CCR7^+^CD45RA^–^) and effector (CCR7^–^CD45RA^–^) memory CD4^+^ T cell frequencies of Treg (FoxP3^+^CD25^+^CD127^–/lo^) CD4^+^ T cells at days 0 (day of vaccination), 14, and 56, measured by mass cytometry (*n* = 9, 3 per M4 and M3M4 group and 3 placebo). In **D**, 1264 is indicated with an open circle. (**C** and **D**) 2-sided, exact Wilcoxon’s signed-rank test. NS, nonsignificant or *P* > 0.05.

We examined if M3/M4/M3M4 vaccinations impacted levels of low-level viremia in plasma measured by SCA and levels of integrated provirus measured by integrated proviral DNA assay (IPDA) (Figure 4, B and C). For SCA analysis, data from participant PID1264 were excluded from analysis because prevaccination increases in SCA were detected ahead of a clinically measured viral blip (153 copies/mL) at day 70 (Figure 4A). While viremia in this participant was unrelated to vaccination, this observation does suggest the utility of SCA for early prediction of HIV-1 reactivation, consistent with a recent report (19). In all other vaccinees, low-level viremia did not change over time, with a median of 0.38 copies/mL (IQR 0.37–0.8 copies/mL) across visits (*n* = 20, *P* > 0.5, days 7, 14, and 70; 2-sided exact Wilcoxon’s signed-rank test) (Figure 4B).

The frequency of total, intact, and defective integrated provirus was measured in 17 vaccinees by IPDA in total CD4^+^ T cells at a single baseline visit and approximately 28 days after vaccination (Figure 4C). Baseline intact and total IPDA exhibited a rank-order positive correlation with baseline SCA values (Figure 3B and Supplemental Figure 4A).

We observed a negative association between baseline total IPDA and fold change response in Mos-1–specific T cells at days 7 and 14, but not Mos-2 T cells (Figure 3B and Supplemental Figure 4A). IPDA measurements were entered as a predictor into a linear regression model with age. After adjusting for age, no independent association was observed between log-transformed baseline IPDA and Mos-1 response to vaccination (Supplemental Table 8).

Following vaccination, the frequency of intact integrated provirus was observed (*n* = 17, *P* = 0.0027, Wilcoxon’s signed-rank test). Notably, the increase within participants was small (average < 10%) and was not supported by measurements of total, 3′ or 5′ defective integrated proviral DNA (all measurements *P* > 0.05; Figure 4C). Increases in intact proviral DNA also did not associate with fold change in the vaccine-elicited T cell responses (Supplemental Figure 4, H and I). With the caveat that our study size is small, we conclude there is limited evidence of a relationship between change in T cell frequency after vaccination and both baseline and fold change in IPDA measurements.

### M3/M4/M3M4 vaccinations did not increase global CD4^+^ T cell activation.

MVA.HIVconsvX vaccines are being tested as part of more complex regimens in ATI studies (e.g., ClinicalTrials.gov NCT06071767). Timing of ATI is influenced by multiple factors. One factor relevant to vaccines is whether vaccination could induce nonspecific CD4^+^ T cell activation, theoretically predisposing a participant to HIV-1 reactivation. To inform design of ATI studies using MVA-vectored vaccines, we examined whether vaccination induced nonspecific changes in CD4^+^ T cell activation (CD38, HLA-DR, CCR5, and PD1), cell cycling (Ki67) in memory subsets, and Treg cell frequencies using mass cytometry. We previously reported that these T cell subsets are stable over time in PWH on longer-term (>5 years) ART (20, 21). PBMCs from 6 vaccine responders who had a >2-fold increase in HIVconsvX-specific T cell frequency at day 14 after vaccination (*n* = 3 M4, *n* = 3 M3M4) and all placebos (*n* = 3) were profiled (Figure 4D). Markers across participants, whether vaccinees or placebo recipients, showed a <2-fold change in percent frequency from prevaccination to either 2 or 8 weeks after vaccination. The low-level viremia detected by SCA prior to the viral blip in PID1264 (Figure 4A) was also not sufficient to impact global activation of circulating memory CD4^+^ T cells. In summary, MVA.HIVconsvX vaccination was not associated with generalized vaccine-induced activation of memory CD4^+^ T cells in the early postvaccination window.

## Discussion

To our knowledge, the M&M Study is the first to test the safety and immunogenicity of the MVA-vectored vaccines M3 and M4 given alone or in combination to PWH on ART. All vaccine regimens, M3, M4, and M3M4, were safe and well tolerated in adult PWH on ART up to 60 years of age.

T cell frequencies, immunodominance, and breadth were measured against both mosaic immunogens at multiple baseline and postvaccination time points in each participant by ex vivo IFN-γ ELISpot assay. Prior to vaccination, all M&M Study participants had ex vivo detectable T cell responses to both the Mos-1 and Mos-2 HIVconsvX immunogens, which, on average, accounted for approximately 50% of the total measured HIV-1–specific T cells.

The M3 and M4 vaccines, given alone or in combination, were comparably immunogenic in PWH on ART; for subsequent analyses, we combined data across the 3 vaccine groups. Eighteen of 21 vaccinees produced a 2- to 24-fold increase in T cell frequencies to one or often both Mos-1 and Mos-2 immunogens. Frequencies of vaccine-induced T cells mostly peaked between 7 and 14 days following vaccination and remained significantly elevated relative to baseline through to 70 days after vaccination. In most vaccinees, postvaccination T cells targeting HIVconsvX regions were comprised of both CD4^+^ and CD8^+^ T cells, with HIVconsvX-specific CD8^+^ T cells coexpressing both IFN-γ and the cytolytic marker CD107a. Ongoing studies will examine the longevity of vaccine-mediated increases in T cell frequencies in participants.

The breadth of HIV-1 T cells targeting conserved HIV-1 proteins has been associated with greater control of HIV-1 viremia in ART-naive PWH (2, 8). In this study, vaccination resulted in increased ex vivo breadth, defined as the number of HIVconsvX subpools measured by the ELISpot assay across vaccination groups. While T cell breadth significantly increased after vaccination, it was unclear whether increased breadth represented de novo induction of HIV-1–specific T cells or expansion of low-frequency memory T cells. To address this question, we increased the sensitivity of our T cell assays by expanding HIVconsvX T cells in a 10-day culture assay prior to testing in ELISpot. Using this approach, almost 40% of putative new subpools were detected prior to vaccination. We conclude that T cells against the remaining subpools were either de novo induced following vaccination or were preexisting but below the limit of detection of the cultured assay used. Whether de novo or preexisting, these newly detected T cell responses were weak and contributed a median of only 2% to the total measured HIVconsvX T cell response across participants following vaccination. We conclude that MVA.HIVconsvX vaccination overwhelmingly increased preexisting or memory HIV-1–specific T cells in PWH on ART.

While MVA.HIVconsvX vaccination poorly primed new T cells in PWH, vaccination successfully redirected HIV-1–specific T cells to highly conserved regions of HIV-1 (22). On average, 70% of postvaccination HIV-1–specific T cells targeted HIVconsvX regions compared with an average of 50% at baseline. This shift in T cell immunodominance toward conserved regions of HIV-1 was maintained over the 10-week study window. Our previous study in which we mapped T cells against OVs in PWH on ART suggested that increased T cell targeting of HIVconsvX regions should also increase T cell targeting of HIV reservoir viruses within participants (9). To examine this, we combined recovery and sequencing of replication competent OVs with detailed T cell epitope mapping in 3 vaccinees. Consistent with prior study, most epitopes mapped against OVs occurred in HIVconsvX regions. Moreover, we confirmed that vaccination with MVA.HIVconsvX regimens expanded T cells targeting epitopes, including sequence variants, in HIV reservoirs.

Recent studies (23, 24) have observed that higher proliferative capacity of HIV-1– or SIV-specific T cells was associated with posttreatment control of HIV or SIV, respectively. In most participants, MVA.HIVconsvX vaccines significantly increased in vivo proliferation of HIV-1–specific T cells in PWH with elevated frequencies maintained for up to 10 weeks. Increased HIV-1–specific T cell in vivo proliferation did not impact global CD4^+^ T cell activation, limiting concerns about MVA vector–associated reactivation of HIV-1–infected cells in ART intervention studies. Altogether, these results support ongoing therapeutic testing of HIVconsvX vaccines in HIV-1 cure approaches to achieve targeted in vivo expansion of T cells in PWH on ART.

Increased HIV-1–specific T cells frequencies resulting from MVA.HIVconsvX vaccination did not impact levels of persistent HIV-1 infection, as measured by either the SCA or IPDA. Notably, SCA measured before vaccination presaged a subsequent plasma viral load blip in 1 study participant, which was unrelated to vaccination demonstrating the sensitivity of this approach. Overall, the absence of an effect of T cell vaccination on the HIV-1 reservoir, here measured over months, was not surprising, given that potential immune-associated changes in the intact HIV-1 reservoir typically occur slowly, over years to decades (25).

Increased age diminishes both humoral and T cell–mediated responses to vaccination, independent of HIV-1 status (26, 27). In our study, age inversely associated with fold change in the T cell responses to both Mos-1 and Mos-2 immunogens from baseline to early and late postvaccination visits. The slope of data (fold change of T cell frequency vs. age) was similar across both peak postvaccination and 10-week time points, suggesting a consistent association between age and vaccine T cell response. This is notable given that the age range of our cohort was 21–60 years, with a median age of 41 years. This median age is young relative to the broader population of PWH in the United States, in which 54% of PWH are estimated to be over 50 years of age (28), although >10 years older than estimated averages in sub-Saharan nations in Africa (29). Altogether, our data suggest that whether a PWH on ART is 20, 30, or 40 years of age, their T cell response to MVA.HIVconsvX vaccination would approximately halve over the subsequent 20 years. We also observed an inverse association between age and the detection of new T cell responses after vaccination, consistent with other studies describing age-associated decreases in naive T cells and vaccine responsiveness in both PWH and people without HIV (18, 27, 30).

Regression analysis also found that after accounting for age, increased time on ART was associated with improved T cell responses to MVA.HIVconsvX vaccination. We and others (21, 31, 32) have reported slow restoration, occurring over years, of multiple parameters of memory T cell function in people initiating ART in chronic HIV-1 infection. Several groups (33–35) have reported that immune aging or inflammaging in PWH, possibly associated with the high prevalence of human cytomegalovirus coinfection, is never fully restored on ART. This contrasts with only minimal T cell dysregulation observed in people who initiate ART in acute HIV-1 (36). We suggest that years of ART is a surrogate measure of immune restoration in people who initiate ART in chronic HIV-1 infection that modulates in vivo proliferation capacity of HIV-1–specific T cells following vaccination. Notably, neither CD4^+^ nadir nor CD4^+^/CD8^+^ ratio associated with T cell response to vaccination, suggesting years of ART may reflect broader changes within participants. Further studies are needed to identify the sweet spot between negative effects of aging and beneficial effects on ART-mediated immune restoration.

Our results build on our recent reports of the M3 and M4 vaccines as a combined boost following vaccination with simian adenovirus–vectored vaccine ChAdOx1.tHIVconsv1 (C1) expressing Mos-1 in people without HIV-1 in the United Kingdom and sites across Kenya, Uganda, and Zambia. In these studies (37, 38), the C1-M3M4 regimen was highly immunogenic, inducing HIVconsvX-specific CD8^+^ T cells capable of inhibiting representative viruses from the 4 major global clades in tissue culture, with some sex- and age-associated effects observed.

The M&M Study has limitations. Likely due to limited study size, we were unable to resolve whether the bivalent mosaic administration of M3M4 provided any advantages over the monovalent vaccines, M3 or M4 alone. Single-vaccine regimens clearly offer benefits over combination regimens in terms of lower complexity and cost. Additional trials are needed to evaluate any advantages of bivalent vaccination regarding targeting diverse HIV-1 reservoirs in PWH. We are particularly interested in whether bivalent immunogens combined with boosting regimens could further increase T cell breadth against HIV variants in the reservoir. Our study size also limited our ability to assess multiple cohort demographics. We were unable to examine the impact of sex and its interaction with age or immune reconstitution on vaccine response. Preexisting monkeypox-specific (mpox-specific) humoral immunity may further limit T cell response to MVA.HIVconsvX vaccines, and given increased mpox vaccination since 2019, baseline MVA-specific antibody titers should be measured in future studies. It is notable that repeated vaccination with recombinant MVA vaccines can successfully boost T cell responses in vaccinees (39), though how HIV-1 infection and increasing age further impact these vaccine responses needs additional study.

In summary, we demonstrate that MVA-vectored vaccines expressing conserved HIVconsvX immunogens, given as monovalent or bivalent mosaics, were safe and strongly immunogenic in PWH on ART. We further show that increased age linearly and negatively correlates with vaccine-induced T cell frequencies. Notably, after adjusting for age, longer time on ART positively associated with Mos-1–specific T cell response to vaccination. Future studies of HIVconsvX and other HIV-1 T cell vaccines in PWH on ART should be designed to consider the confounding effects of both residual HIV-1 immune dysregulation and participant age.

## Methods

### Sex as a biological variable.

This study included both men and women. Sex is detailed in both safety and immunogenicity data.

### M&M cohort.

The study was a double blind, placebo-controlled study in which 24 PWH were randomly assigned to 1 of 4 arms and received vaccination with M3, M4, M3M4, or placebo at a 7:7:7:3 ratio using a block randomization design. Vaccinees received a single intramuscular vaccination of either M3, M4, or M3M4 at a total dose of 2 × 10^8^ PFU on day 0 (Table 1). Whole blood or leukapheresis was collected from each participant at each in-person visit, with a final sample collection visit at 70 days after vaccination.

Participants were ≥ 18 years of age (21 male, 3 female) with documented HIV-1 infection (Table 1), standard-of-care ART, plasma HIV-1 RNA < 50 copies/mL for 24 months, and a CD4^+^ cell count ≥ 350 cells/mm^3^ at enrollment. Exclusion criteria comprised prior receipt of any HIV-1 vaccine, recombinant adenovirus, or MVA vaccine; immunomodulatory medications within 90 days of screening; or a history of autoimmune disease.

Participants had 1–3 baseline visits (median = 3) including day of vaccination designated as day 0, followed by study visits at days 7, 14, 28, 56, and 70, with telephone assessments at days 84 and 168. Whole blood was collected at all visits through day 70. HIV-1 RNA was measured in all participants at screening, at days 7 and 70, and at specified interim visits.

### Vaccines.

The 2 HIVconsvX vaccine mosaics were designed in silico using full-genome sequences of group M HIV-1 in the Los Alamos National Laboratory HIV Sequence Database, as of approximately September 2013 (8). The HIVconsv3 immunogen was generated using Mos-1 sequences, and HIVconsv4 immunogen was generated using Mos-2 sequences (Figure 1A). These immunogens were inserted into replication-deficient poxvirus MVA to generate the M3 and M4 vaccines, respectively (8). Vaccines were manufactured at IDT Biologika.

### COVID-19 and mpox.

M&M vaccinations overlapped with the 2021 US COVID-19 pandemic and subsequent COVID-19 vaccinations but occurred prior to the 2022 US mpox outbreak and mpox vaccination of at-risk individuals. One participant was discontinued from study procedures due to undisclosed receipt of a COVID-19 vaccination within 14 days of enrollment, constituting an enrollment violation. This participant was replaced but followed up on for safety assessments.

### PBMC isolation.

PBMCs were isolated from blood and apheresis products within 6 hours of collection. Blood was centrifuged at 900*g* for 15 min at room temperature (RT), and the plasma was collected and centrifuged a second time at 1,400*g* for 15 min at RT before freezing. Blood products were diluted 1:2.5 in 2% FBS/PBS, overlaid onto 15 mL of Ficoll-Plaque in Sepmate PBMC isolation tubes (StemCell), and spun at 1,200*g* for 20 min at RT. PBMCs were collected, washed, counted, and frozen (13). Apheresis products were similarly diluted and PBMCs isolated following centrifugation.

### Cell culture.

Two culture media were used, R10+ (RPMI, 10% FBS, 10 mM HEPES, 2 mM l-glutamine, 1 mM sodium pyruvate, and penicillin-streptomycin) and RAB10 (FBS as replaced with 10% human AB serum).

### Peptides.

T cell functions were measured against peptides spanning the 6 HIVconsvX regions pooled into Mos-1 and Mos-2 (18-mer peptides overlapping by 11 amino acids; 119 peptides in each pool), junctional sequences between each HIV-1 region (18-mers, 10 peptides), HIV-1 clade B ENV (18-mers, 113 peptides), and clade B NEF/ACC pool (18-mers, 95 peptides). ACC describes peptides spanning HIV clade B Tat, Rev, Vif, Vpu, and Vpr. To examine the T cell breadth, all Mos-1 and Mos-2 peptides were also split into 5 subpools (38–64 peptides/subpool), labeled A–E (Figure 1A). Overlapping peptides were also synthesized to match autologous virus sequences within participants and tested individually (10 μg/mL) in ex vivo ELISpot, as previously described (9). Optimal CD8^+^ T cell peptides were predicted using Los Alamos National Laboratory’s epitope location finder, which incorporates HLA prediction software, and confirmed empirically. An epitope was defined as optimal if its measured frequency was greater than or equal to its parent 18-mer peptide (40).

### Ex vivo ELISpot.

Cryopreserved PBMCs were thawed, rested overnight, and added to precoated and blocked IFN-γ ELISpot plates (MabTech) at 0.25 × 10^6^ PBMCs/well. Controls included 6 negative mock control (medium-only) and 2 mitogen (5 μg/mL phytohemagglutinin [PHA]) wells. Peptides, previously aliquoted at 2× concentration and stored at –80°C, were added in quadruplicate to the plate with a final assay concentration of 1 μg/mL and incubated for 18–20 hours at 37°C, 5% CO_2_. Stability of peptide plates was assessed yearly against reference PBMCs. Coating, development, and reading of ELISpot plates (AID Reader ELR081512367; Autoimmun Diagnostika GmbH) have been described previously (13). Spots were enumerated using a standard counting setting, producing a frequency of spot-forming units (SFUs) per total input cells. Data were submitted for independent statistical review prior to unblinding. T cell responses were considered specific if (a) the (average of peptide-stimulated wells) – (average of mock wells) was > 20 SFUs/10^6^ PBMCs; (b) the (average of peptide-stimulated wells) was ≥ 4× the (average of mock wells); and (c) peptide-stimulated wells did not contain any zero values. To enable calculation of log_2_ fold change (log_2_FC), if the average of the mock-subtracted wells was negative, then a value of 20 SFUs/10^6^ PBMCs was used. Also, for FC calculations, the baseline value, B, was calculated as the mean of 1–3 (median = 3) prevaccination time points, mostly between days –85 and 0, the day of vaccination. For 1 participant, 937, only 1 prevaccination time point was available. A new T cell response to vaccination was defined as (a) positive to a peptide subpool at ≥ 2 postvaccination visits but (b) negative at baseline time points. Full (6-digit) HLA class-Ia typing was performed using PacBio sequencing of DNA from whole blood (Histogenetics).

### ICS.

Antibody staining and permeabilization was performed as previously described (11) and antibodies/fluorophores are listed in Supplemental Table 9. ICS was performed with the general panel (IFN-γ–PE, CD107a-APC) for all vaccine recipients (*n* = 21). For each stain, 1 × 10^6^ PBMCs were incubated with no peptide (0.58% DMSO), a final concentration of 2 μg/mL Mos-1 or Mos-2 peptide pool, or 3–5 μg/mL PHA. Fluorescence minus one controls were used for gating of functional markers (Supplemental Figure 5). Samples were acquired on a BD LSRFortessa (BD Biosciences) and analyzed using FlowJo (version X10.8.1). Compensation controls were prepared for each fluorochrome using anti-mouse Ig, κ compensation particles (552843, BDTM CompBead). On average, >325,000 CD3^+^ events/sample were acquired for analysis. A positive functional response (IFN-γ^+^ or IFN-γ^+^CD107a^+^) in Mos-1 or Mos-2 stimulated CD4^+^ or CD8^+^ T cells was defined as (a) ≥2-fold mock-stimulated cells and (b) (events in the functional gate of the peptide-stimulated sample) – (events in the functional gate of the mock-stimulated sample) ≥ 25. If a sample did not meet this threshold, then a zero value was assigned (Supplemental Table 6).

### STCLs and ELISpot.

After overnight thawing and rest, 2 × 10^6^ PBMCs/mL from a baseline visit were stimulated with either Mos-1 or Mos-2 peptide pool (1 μg/mL, 128–171 peptides/pool) and cultured for 10 days in RPMI supplemented with 10% AB serum, with addition of IL-2 (1,000 U/mL) on days 3 and 7, with additional feeding between days 8 and 10 as needed. On day 10, cells were washed 3 times then rested for 30–36 hours (13). Cells were stimulated with a peptide subpool A (2 μg/mL, detected in ex vivo ELISpot in all participants tested), selected peptide subpools B–E (2 μg/mL, quadruplicate, 40,000 cells/well), mock (quadruplicate), or PHA (10 μg/mL, positive control) for 18–20 hours. ELISpots were developed as per ex vivo ELISpot (as described above). Specific T cell responses were calculated as follows: (average of peptide stimulated wells) – (average of mock replicates), expressed per 10^6^ cells. Positivity criteria for stimulated wells were defined as (a) background-subtracted SFUs > 200 SFUs/10^6^ cells, (b) average SFUs of peptide-stimulated wells ≥ 3 times the average SFUs of mock wells, and (c) peptide-stimulated wells contain no zero values. As an internal control for HIV-specific T cell expansion in each STCL, we also required ≥3.5-fold increase in the subpool A stimulated wells relative to the measured ex vivo T cell frequency (Supplemental Table 5).

### Mass cytometry.

PBMCs were phenotyped by mass cytometry. The 32-marker panel, staining, acquisition (700,000 events acquired), analysis conditions, and gating strategy have been detailed previously (21); metal-tagged antibodies are listed in Supplemental Table 9. Controls included unstained cells, cells stained with lineage markers (21), and barcoded reference samples spiked into all samples.

### Viral outgrowth assays and sequencing.

For PID749 and PID1095, virus outgrowth and 5′ and 3′ half sequencing of individual viruses was as previously reported (9). For PID371, virus outgrowth was performed by Southern Research as described previously (41). Briefly, this method culture isolated CD4^+^ T cells with a cytokine milieu (25 ng/mL each of TNF-α, IL-6, IL-7, IL-10, and IL-15) for 7 days prior to pan–T cell activation at day 0. Culture supernatants were collected on day 12 and frozen. HIV outgrowth was confirmed by p24 ELISA (PerkinElmer). Extraction of viral RNA and sequencing were performed as described previously (9). All sequences have been deposited in GenBank, as detailed in Supplemental Table 9.

### HIV-1 IPDA.

The IPDA was performed on CD4^+^ T cells isolated from cryopreserved PBMCs by Accelevir Diagnostics under company standard operating procedures (42, 43).

### Plasma HIV-1 RNA quantification.

Persistent plasma viremia was measured in 5 mL of double-spun EDTA plasma using the automated SCA. Automated SCA is an adaptation of the Hologic Panther Aptima HIV-1 Viral Load test modified to test 9 replicates of the same sample that are algorithmically interpreted to derive results down to 0.4 copies of HIV-1 RNA/mL of plasma (44, 45). Assay limit of detection (LOD) was 0.38 copies/mL plasma; accordingly, all measurements < LOD were assigned a value of 0.38.

### Key reagents.

Key reagents, resources, sources, and relevant identifiers are detailed in Supplemental Table 9.

### Study design.

This sample size was designed such that the probability of zero grade 3 or higher safety events through 28 days following vaccination for *n* = 7 (each vaccine group) and *n* = 21 (combined vaccine groups) would be 35% and 13%, respectively (upper limit of 95% 1-sided exact binomial confidence limited). This study size provided sufficient power of at least 80% to detect a log_2_FC in T cell frequency in groups sizes *n* = 7. Power calculations were informed using baseline T cell ELISpot data from previous observational studies that observed within-individual variation <16.5% coefficient of variation for weekly and monthly sampling (9, 46). The placebo group was included to maintain blinding during immunization and AE reporting. No comparisons between placebo recipients and vaccinees were planned for safety or immunogenicity reporting.

Each study visit had an assigned study window. For analyses at the group level (e.g., M4 vaccinees), participant visits falling within the study window were assigned that visit day.

### Statistics.

Statistical analyses were performed and visualizations produced using GraphPad Prism v9 and R v4.5.2 (47), and frequency tables for AE reporting were generated using SAS Software v9.4 (SAS Institute). Fold changes were calculated as the postbaseline value divided by the baseline value, with baseline defined as the average of all measurements prior to the first vaccination. All tests were 2 sided and assumed a type 1 error rate of α = 0.05. Within-group comparisons were performed using exact Wilcoxon’s signed-rank tests, and between-group comparisons used exact Wilcoxon’s rank-sum tests. Correlation was reported as Spearman’s rank-order coefficients with corresponding ρ and *P* values, except when diagnostic checks (such as assessment of linearity, approximate normality of the variables, and absence of influential outliers) supported the use of Pearson’s correlation. As prespecified in the analysis plan, no adjustments for multiple comparisons were applied given the exploratory nature of this phase I study and its small sample size. Operators were blinded to vaccination status for ELISpot, SCA, and IPDA analyses. ICS, generation of STCLs, and mass cytometry were performed following unblinding.

### Study approval.

All participants were enrolled through the University of North Carolina Institute for Global Health and Infectious Diseases Clinical Trials Unit (IGHID CTU). The study was approved by the local Institutional Biomedical Review Board (IRB 18-250) and performed under IND18368. Written informed consent was received prior to enrollment.

### Data availability.

Safety data are reported on ClinicalTrials.gov, NCT03844386. Participant-level data listings for most results are provided in the supplemental materials, with all primary data tabulated in the Supporting Data Values file. HIV sequences were deposited in GenBank (PRJNA666896, MT307344–MT308415, and MW054719–MW054856). Other data are available from the corresponding author upon reasonable request.

## Author contributions

NG conceived, designed, and holds the investigational new drug (IND) application for this study with contributions from TH, CLG, and YX. CLG, AMKW, JDK, CEB, SMP, LB, AC, EGTW, TH, JJE, MGH, LF, and NG wrote the clinical study protocol and were responsible for regulatory submissions, including safety monitoring committee coordination. AMKW, CK, and MGH were responsible for randomization and analysis of study data. TH created the HIVconsvX vaccines. CLG, JDK, ABB, CEB, SMP, LB, JJE, and DMM contributed to clinical oversight of the study procedures and clinical review of study data. YX, FRS, SZC, SAM, SS, SK, ATS, JAW, GTC, and MAF generated and analyzed immunological data. MJM, GML, JCC, JWM, and SZ generated and analyzed virologic data. NG, CLG, TH, YX, and AMKW wrote the primary manuscript with revisions from all coauthors. Co–first authors CLG and YX are listed in alphabetical order.

## Conflict of interest

TH is named as an inventor on the HIVconsvX immunogen patent protected under EP14846993.5 and PCT/US14/58422 (WO2015048785). AMKW holds shares in Eli Lilly and Company.

## Funding support

This work is the result of NIH funding, in whole or in part, and is subject to the NIH Public Access Policy. Through acceptance of this federal funding, the NIH has been given a right to make the work publicly available in PubMed Central.

- NIH NIAID grant U01AI131310 (to NG, clinical trial).
- NIH NIAID grants HHSN272201100021I/HHSN27200037, subcontract OX-14007.004.0037-212 (to TH, C62 vaccine).
- International AIDS Vaccine Initiative, through USAID and other donors, available at http://www.iavi.org (to TH, M3 vaccine).
- European and Developing Countries Clinical Trials Partnership SRIA2015-1066 (to TH, C1 and M4 vaccines).
- European Commission’s Horizon 2020 Research and Innovation Programme 681137 (to TH, M4 vaccine).
- NIH NIAID grant UM1TR004406 to North Carolina Translational and Clinical Sciences Institute.
- NIH NIAID grant P30AI050410 to UNC Center for AIDS Research.
- NIH NIAID grant P30CA016086 to UNC Mass Cytometry & Cell Omics Core.

## Supplementary Material

Supplemental data

ICMJE disclosure forms

Supporting data values

## Figures and Tables

**Table 1:**
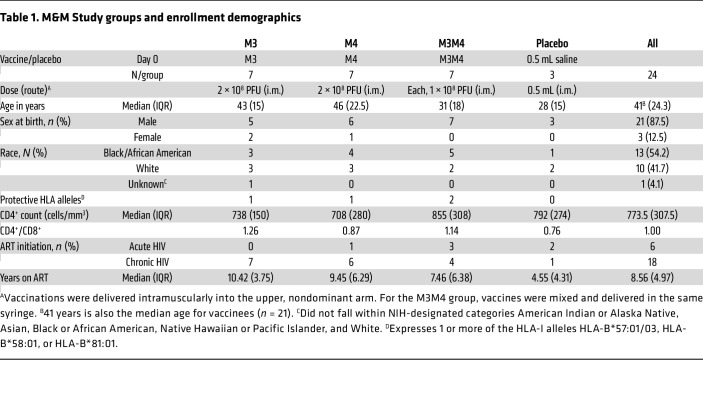
M&M Study groups and enrollment demographics

**Table 2:**
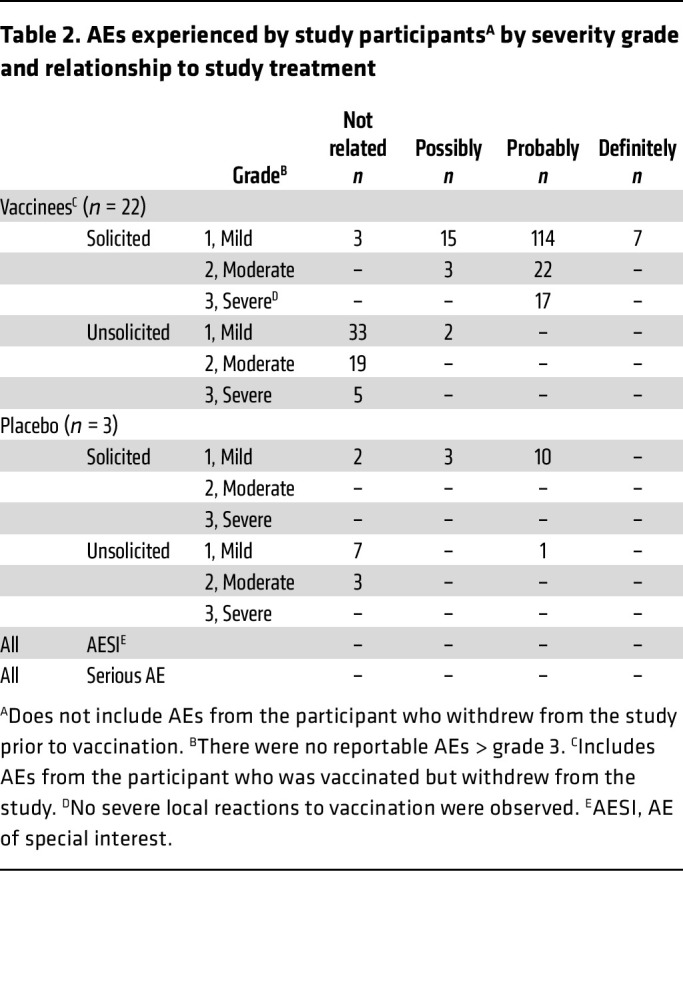
AEs experienced by study participants^A^ by severity grade and relationship to study treatment

**Table 3:**
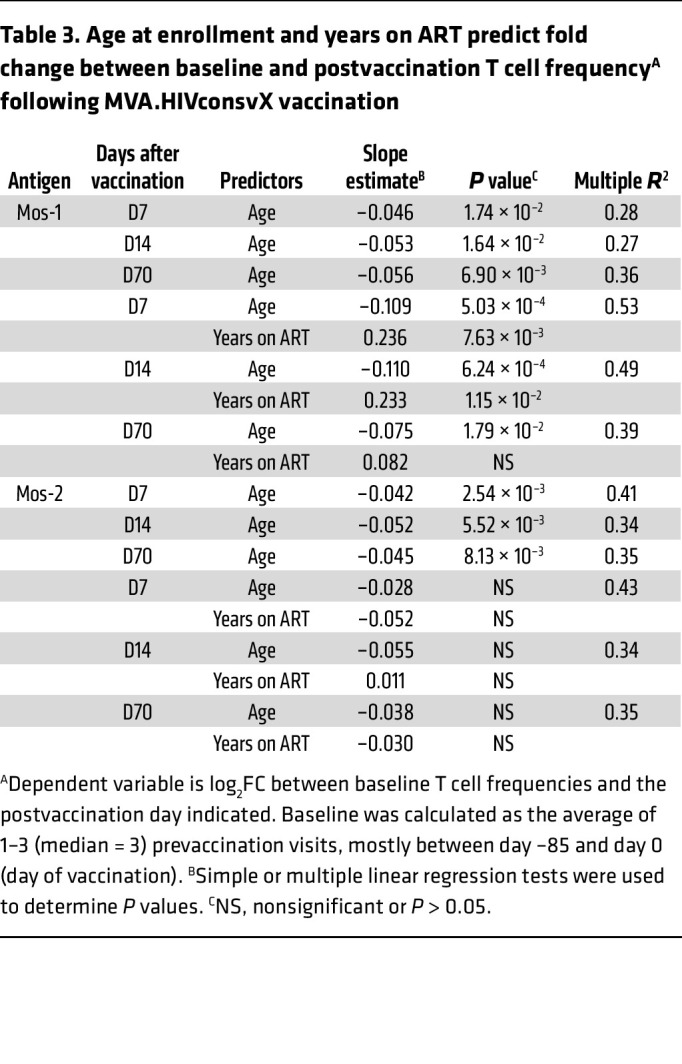
Age at enrollment and years on ART predict fold change between baseline and postvaccination T cell frequency^A^ following MVA.HIVconsvX vaccination

## Version 1 05/05/2026

In-Press Preview

## Version 2 06/15/2026

Electronic publication
