# Supplementary material for: MVA.HIVconsvX vaccination–evoked T cell expansion inversely associates with age in people with HIV-1 on antiretroviral therapy
Source: J Clin Invest. 2026 May 5;136(12):e193547. doi: 10.1172/JCI193547 (PMC13262733; doi:10.1172/JCI193547)

Supplementary Table 1: M&M Individual Participant Demographics

Study Arm	Participant	Sex at birth (Male M, Female F)	Race[1]	Ethnicity[2]	HLA Protective Alleles[3]	CD4 Nadir	Enrollment data					
							Age (yr)	ART (yr)	CD4 count (/mm³)	CD8 count (/mm³)	CD4: CD8	VL (copies/ mL)
**M3**	00836	M	AA	NH			33	7.83	758	794	0.95	20
	01237a	M	U	H			41	10.17	642	766	0.84	20
	01249	M	C	NH			28	8.11	738	585	1.26	20
	00937	F	AA	NH			48	9.21	1235	976	1.27	40
	00835	M	C	NH			43	9.61	789	589	1.34	20
	01214	M	C	NH			57	16.14	605	464	1.3	20
	01009	F	AA	NH	B*58:01		60	11.86	515	555	0.93	20
					Average		44.28	10.42	754.57	675.57	1.23	
**M4**	00749[4,5]	M	AA	NH			30	5.87	484	332	1.46	20
	01262	M	C	H			55	12.16	708	938	0.75	20
	01264[5,6]	M	AA	NH			37	6.03	1002	745	1.3	20[6]
	01276	M	AA	NH			46	11.12	427	493	0.87	20
	01277[5]	M	AA	NH	B*81:01		31	2.47	795	659	1.2	40
	01095[4]	F	C	NH			58	10.41	571	867	0.66	20
	**00115**	M	C	NH			59	18.12	821	996	0.82	40
					Average		45.14	9.45	686.86	718.57	1.01	
**M3M4**	**00631**[5]	M	AA	NH			29	6.44	944	648	1.46	20
	**00371**[4]	M	AA	NH	B*57:03, 58:01		30	10.72	566	538	1.05	20
	01153	M	C	NH			55	12.71	847	942	0.9	40
	01280	M	C	NH			54	6.55	1086	616	1.76	40
	**01213**	M[#]	AA	NH			21	2.45	463	406	1.14	20
	01295[5]	M	AA	NH	B*57:03		41	9.01	1365	876	1.56	20
	01293[5]	M	AA	NH			31	4.34	855	1219	0.7	20
					Average		37.29	7.46	875.14	749.29	1.22	
**Placebo**	00818[5]	M	B/AA	NH			24	6.68	792	1447	0.55	20
	01270[5]	M	C	NH			28	4.59	1070	1322	0.81	20
	01220[5]	M	C	NH			52	2.37	522	787	0.66	20
					Average		34.67	4.55	794.67	1185.33	0.67	
					Cohort Average (n=24)		41.29	8.54	775.00	773.33	1.06	

[1]C: White/Caucasian, AA: Black/African American, U: Did not fall in NIH categories of American Indian or Alaska Native, Asian, Black or African American, Native Hawaiian or Other Pacific Islander, or White
[2]H: Hispanic, NH: Non-Hispanic
[3] Participant expresses one or more of HLA I alleles, B*57:01/03, B*58:01, B*81:01
[4] T-cell response against HIV outgrowth virus/s measured
[5]Phenotyped by mass cytometry to assess post-vaccination T-cell activation detailed Figure 4D.
[6] Virus blip at Day 70 (153 copies/mL), Day 86 (86 copies/mL) detailed Figure 4B
Bolded Participant Identifiers: initiated ART in acute HIV-1 infection, # PID01213 is a transgender woman.

Supplementary Table 2: Number of all solicited[1] and reportable unsolicited adverse events (including recurrences) by treatment arm.

Adverse Event	Treatment Arm<br>M3 (n=7)	M4 (n=7)	M3M4 (n=8[1])	Placebo (n=3)	Total
**Solicited**					
Injection Site Pain and, or tenderness	13	12	13	3	41
Headache	9	4	5	3	21
Fatigue	7	5	5	2	19
Malaise	9	5	3	2	19
Myalgia	5	7	4	1	17
Influenza Like Illness	7	2	4	1	14
Chills	4	4	3	0	11
Decreased Appetite	4	1	3	1	9
Nausea	4	1	4	0	9
Arthralgia	3	0	3	1	7
Hyperhidrosis	3	1	1	1	6
Dizziness	4	1	0	0	5
Abdominal Pain	1	0	2	0	3
Body Temperature Increased	2	1	0	0	3
Injection Site Swelling	3	0	0	0	3
Injection Site Warmth	0	1	2	0	3
Vomiting	1	0	1	0	2
Diarrhea	0	1	0	0	1
Injection Site Discoloration	0	1	0	0	1
Injection Site Erythema	0	0	1	0	1
Injection Site Induration	0	0	1	0	1
**Unsolicited**					
Procedural Hypertension	12	13	15	5	45
Blood Pressure Increased	0	3	1	0	4
Tissue Infiltration	0	1	3	0	4
Citrate Toxicity	1	0	2	0	3
Presyncope	1	0	0	1	2
Blood Creatinine Increased	1	0	0	0	1
Blood HIV RNA Increased	0	1	0	0	1
Erythema	0	1	0	0	1
Hyperhidrosis	1	0	0	0	1
Post Procedural Contusion	1	0	0	0	1
Post Procedural Hematoma	0	1	0	0	1
Procedural Dizziness	0	0	0	1	1
Procedural Pain	0	0	1	0	1
Pyrexia	0	0	0	1	1
Vessel Puncture Site Hemorrhage	0	0	0	1	1
Vessel Puncture Site Pain	0	0	0	1	1
**Total**	96	67	77	25	265

[1] In the M3M4 group, one participant was vaccinated with M3M4 and completed study visits but had received a COVID-19 vaccination within two weeks of enrollment. This participant was subsequently replaced.

Supplementary Table 3: Fold-change in Mos-1- and Mos-2-specific T-cell frequencies following vaccination measured by IFN-γ ex vivo ELISpot

Study Arm	Participant	Sex[1]	Age[2]	Years on ART[3]	Protective HLA Alleles[4]	Fold change from Baseline[5]					
						Day 7		Day 14		Day 70	
						Mos-1	Mos-2	Mos-1	Mos-2	Mos-1	Mos-2
**M3**	00836	M	33	7.83		8.877	6.364	8.282	6.277	7.835	5.885
	01237a	M	41	10.17		2.789	2.657	2.770	3.053	1.434	1.197
	01249	M	28	8.11		9.849	2.908	24.590	6.916	20.966	6.543
	00937	F	48	9.21		4.959	3.681	6.727	4.993	3.031	2.204
	00835	M	43	9.61		2.848	2.129	4.857	5.169	2.868	2.114
	01214	M	57	16.14		2.000	1.495	1.444	1.021	1.275	1.027
	01009	F	60	11.86	B*58:01	1.537	1.028	1.434	1.057	1.357	1.102
**M4**	00749	M	30	5.87		5.242	5.856	4.959	5.736	3.605	3.482
	01262	M	55	12.16		2.114	2.219	2.828	3.182	2.888	2.497
	01264	M	37	6.03		1.007	2.282	1.266	4.347	1.079	2.694
	01276	M	46	11.12		1.569	1.892	1.395	1.778	1.125	1.310
	01277	M	31	2.47	B*81:01	1.516	9.190	2.000	13.929	ND[6]	ND
	01095	F	58	10.41		1.181	1.602	0.993	1.181	ND	ND
	**00115**	M	59	18.12		3.272	1.905	7.311	4.500	1.932	1.516
**M3M4**	**00631**	M	29	6.44		11.472	3.138	8.340	2.000	4.287	1.357
	**00371**	M	30	10.72	B*57:03, 58:01	7.674	3.364	3.864	2.549	2.189	1.892
	01153	M	55	12.71		3.364	5.897	3.482	7.062	2.532	4.627
	01280	M	54	6.55		ND	ND	0.722	0.883	0.993	1.165
	**01213**	M#	21	2.45		6.021	6.589	8.340	9.781	7.062	8.225
	01295	M	41	9.01	B*57:03	1.625	2.657	1.892	3.117	1.395	1.548
	01293	M	31	4.34		2.000	2.585	2.770	4.228	2.732	4.112
**Placebo**	00818	M	24	6.68		0.889	0.847	1.102	1.102	1.395	0.883
	01270	M	28	4.59		1.057	1.064	0.737	0.920	0.920	0.514
	01220	M	52	2.37		1.050	0.973	1.464	1.414	0.883	1.028
		M3, M4, M3M4 Vaccinees only: Median (IQR)				2.819 (3.579)	2.657 (1.848)	2.828 (3.987)	4.228 (4.013)	2.532 (1.721)	2.114 (2.121)
		Vaccinees with Non-Protective Alleles: Median (IQR)				3.060 (1.824)	2.621 (1.687)	3.482 (2.956)	4.347 (3.596)	2.800 (1.593)	2.350 (1.530)
		Vaccinees with Protective Alleles: Median (IQR)				1.581 (1.842)	3.010 (1.587)	1.946 (2.526)	2.833 (3.068)	1.395 (1.525)	1.548 (1.530)

[1] Sex at birth, M = male, F = female
[2] age (years) enrollment
[3] years from antiretroviral therapy (ART) initiation to enrollment
[4] Participant expresses one or more of the following HLA I alleles, B*57:01/03, B*58:01, B*81:01
[5] Fold-change from the average of two pre-vaccination timepoints to indicated visit (see Methods)
[6] ND – not done. No sample available for testing.
Bolded Participant Identifiers: initiated ART in acute HIV-1 infection # PID01213 is a transgender woman.

Supplementary Table 4: Detection of baseline HIVconsvX-specific T-cells following *in vitro* culture.

		Ex vivo ELISpot			Short term cell lines (STCL) then ELISpot			
Participant	Study Group	Sub-pool tested by ELISpot	Baseline (SFU/10^6 PBMC)[1]	Post-vaccination (SFU/10^6 PBMC)[2]	Stimulating Peptide Pool	Sub-pool tested by ELISpot	Day -28 (SFU/10^6 STCL)	Fold-expansion relative to baseline ex vivo frequency[3]
**836**	**M3**	A (G1)	225	1180	Mos-1	**A (G1)**	**2200**	**9.8**
		C (P3-A)	≤20	69		C (P3-A)	≤20	-
		E (P6)	≤20	71		E (P6)	≤20	-
					Mos-2	**A (G1)**	**2460**	**11**
						**C (P3-A)**	**469**	**23.4**
						E (P6)	≤20	-
1237a	M3	A (G1)	106	300	Mos-1	A (G1)	329	3.1
		C (P3-A)	≤20	27		C (P3-A)	≤20	-
					Mos-2	A (G1)	≤20	-
						C (P3-A)	≤20	-
**1249**	**M3**	A (G1)	45	209	Mos-1	**A (G1)**	**700**	**15.5**
		C (P3-A)	≤20	61		C (P3-A)	≤20	-
		D (P3-B)	≤20	105		D (P3-B)	≤20	-
		E (P6)	≤20	143		**E (P6)**	**794**	**39.7**
					Mos-2	**A (G1)**	**1065**	**24**
						C (P3-A)	≤20	-
						**D (P3-B)**	**300**	**15**
						E (P6)	≤20	-
937	M3	A (G1)	76	451	Mos-1	A (G1)	≤20	-
		C (P3-A)	≤20	280		C (P3-A)	≤20	-
		E (P6)	≤20	178		E (P6)	≤20	-
					Mos-2	A (G1)	≤20	-
						C (P3-A)	≤20	-
						E (P6)	≤20	-
**835**	**M3**	A (G1)	219	513	Mos-1	**A (G1)**	**3983**	**18.2**
		E (P6)	≤20	160		**E (P6)**	**794**	**39.7**
					Mos-2	**A (G1)**	**1827**	**8.3**
						**E (P6)**	**725**	**36.3**
**1214**	**M3**	A (G1)	388	612	Mos-1	**A (G1)**	**8700**	**22.4**
		C (P3-A)	≤20	22		C (P3-A)	≤20	-
					Mos-2	**A (G1)**	**8508**	**22**
						C (P3-A)	150	7.5
1009	M3	A (G1)	1682	1731	Mos-1	A (G1)	1208	-
		C (P3-A)	≤20	45		C (P3-A)	≤20	-
		E (P6)	≤20	71		E (P6)	≤20	-
					Mos-2	A (G1)	1894	1.1
						C (P3-A)	≤20	-
						E (P6)	≤20	-
**749**	**M4**	A (G1)		288	Mos-1	A (G1)	≤20	-
		C (P3-A)		85		C (P3-A)	≤20	-
		D (P3-B)		212		D (P3-B)	≤20	-
					Mos-2	**A (G1)**	**200**	**8.7**
						C (P3-A)	≤20	-
						D (P3-B)	142	7.1
**1262**	**M4**	A (G1)	150	304	Mos-1	**A (G1)**	**1529**	**10.2**
		D (P3-B)	≤20	32		**D (P3-B)**	**644**	**32.2**
					Mos-2	**A (G1)**	**1479**	**9.9**
						**D (P3-B)**	**300**	**15.0**
**1264**	**M4**	A (G1)	296	374	Mos-1	A (G1)	≤20	-
		C (P3-A)	≤20	78		C (P3-A)	≤20	-

		D (P3-B)	≤20	105		D (P3-B)	≤20	-
					Mos-2	**A (G1)**	**3225**	**10.9**
						C (P3-A)	≤20	-
						D (P3-B)	≤20	-
1277	M4	A (G1)	859	1295	Mos-1	A (G1)	≤20	-
		D (P3-B)	≤20[1]	275		D (P3-B)	≤20	-
		E (P6)	≤20[1]	164		E (P6)	≤20	-
					Mos-2	A (G1)	≤20	-
						D (P3-B)	≤20	-
						E (P6)	≤20	-
**1095**	**M4**	A (G1)[1]	348	759	Mos-1	**A (G1)**	**2771**	**8.0**
		C (P3-A)	≤20	118		C (P3-A)	≤20	-
		E (P6)	≤20	198		**E (P6)**	**1113**	**55.6**
					Mos-2	**A (G1)**	**2260**	**6.5**
						C (P3-A)	≤20	-
						**E (P6)**	**1760**	**88.0**
**631**	**M3M4**	A (G1)	91	645	Mos-1	A (G1)	≤20	-
		B (G2+P4+P5)	≤20	263		B (G2+P4+P5)	≤20	-
		C (P3-A)	≤20	67		C (P3-A)	≤20	-
		D (P3-B)	≤20	116		D (P3-B)	≤20	-
		E (P6)	≤20	125		E (P6)	≤20	-
					Mos-2	**A (G1)**	**1656**	**18.2**
						B (G2+P4+P5)	≤20	-
						C (P3-A)	≤20	-
						D (P3-B)	≤20	-
						E (P6)	≤20	-
**371**	**M3M4**	A (G1)	759	2498	Mos-1	A (G1)	2331	3.1
		B (G2+P4+P5)	≤20	144		B (G2+P4+P5)	≤20	-
					**Mos-2**	**A (G1)**	**2929**	**3.9**
						B (G2+P4+P5)	≤20	-
**1153**	**M3M4**	A (G1)	46	192	Mos-1	**A (G1)**	**250**	**5.4**
		E (P6)	≤20	50		**E (P6)**	**213**	**10.6**
					Mos-2	**A (G1)**	**310**	**6.7**
						E (P6)	≤20	-
**1213**	**M3M4**	A (G1)	81	414	Mos-1	**A (G1)**	**1581**	**19.5**
		C (P3-A)	≤20	92		C (P3-A)	≤20	-
					Mos-2	**A (G1)**	**1825**	**22.5**
						C (P3-A)	≤20	-
**1295**	**M3M4**	A (G1)	153	401 (D14)	Mos-1	**A (G1)**	**3135**	**20.5**
		B (G2+P4+P5)	≤20	48 (D14)		B (G2+P4+P5)	≤20	-
		D (P3-B)	≤20	27 (D14)[6]		D (P3-B)	≤20	-
					Mos-2	**A (G1)**	**3129**	**20.5**
						B (G2+P4+P5)	≤20	-
						D (P3-B)	≤20	-
**1293**	**M3M4**	A (G1)	645	2113 (D14)	Mos-1	**A (G1)**	**11223**	**17.4**
		C (P3-A)	≤20	73 (D14)[5]		C (P3-A)	94	4.7
					Mos-2	**A (G1)**	**12567**	**19.5**
						**C (P3-A)**	**369**	**18.4**

[1] Baseline T-cell frequency is the average of two pre-vaccination visits. [2] Day 7 post-MVA.HIVconsvX visit unless otherwise specified in parentheses. Note: A HIVconsvX sub-pool was assigned positive post-vaccination only if positivity criteria for ex vivo ELISpot (Methods) were met at 2 or more post-vaccination visits. [3] Fold-change calculated from baseline T-cell frequency (the average of two pre-vaccination visits, details Methods). For sub-pools <20 SFU/10^6 PBMC and/or had a replicate value of '0', a value of 20 SFU was assigned to calculate fold change. [4] In all participants, a positive ex vivo T-cell response (details Methods) was detected against sub-pool A, G1. [5] PID1293, T-cell response in ex vivo ELISpot to sub-pool C was negative at Day 7 but positive at Day 14 (shown) [6] PID1295, T-cell response in ex vivo ELISpot to sub-pool D was negative at Day 7 but positive at Day 14 (shown). **Bold** = Positive HIV-specific T-cell response to HIVconsvX in cultured ELISpot. Criteria: > 200 SFU/10^6 cells, > 3-fold above mock-stimulated cells. For sub-pools B-E, a > 3.5-fold expansion to sub-pool A (G1) in the same parent STCL was required as a positive control for HIV-specific expansion of T-cells.

Supplementary Table 5: T-cell frequencies measured by ex vivo IFN-γ ELISpot against autologous HIV sequences.

Participant - group	HXB2 Region	Autologous Virus Sequence (predicted HLA restriction) [1]	# autologous viruses in which sequence occurs	Sequence in HIVCONSX immunogen[2] (Y/N)	%Seq. Identity with Mos-1 peptide[2]	%Seq. Identity with Mos-2 peptide[2]	%Seq. Identity with Clade B peptide[3]	Ex vivo ELISpot SFU/10^6, (SEM) [4]	
								Pre-vaccination Visit 3 - Baseline	Post-MVA.HIVconsvX Vaccination Visit 8 - Day 28
00371-M3M4	Gag 240→249	TSTLQEQIGW[4] (HLA-B*57:03, B*58:01)	3/3	Y	90.00%	100.00%	100.00%	321 (21)	882 (10)
	Gag 244→261	QEQIGWMTNNPPIPVGEI	3/3	Y	88.88%	100.00%	100.00%	276 15)	875 (31)
	Gag 300→317	FYKTLRAEQASQDVKNWM	3/3	Y	94.44%	83.33%	94.44%	23 (3)	13 (5)
	Gag 196→213	AAMQMLKETINEEAAEWD	3/3	Y	94.44%	100.00%	100.00%	21 (4)	12 (3)
	Pol 873→890	LKKIIGQVRDQAEHLKTA	3/3	Y	94.44%	100.00%	100.00%	38 (8)	75 (3)
00749-M4	Pol 481→498	IAEIQKQGQGQWTYQIYQ	15/15	Y	94.44%	100.00%	100.00%	146 (15)	395 (23)
	Pol 929→946	QKQISKIQNFRVYYRDNR	15/15	Y	88.88%	88.88%	88.88%	60 (9)	183 (12)
	Pol 969→986	NSEIKVVPRRKAKIIRDY	15/15	Y	88.88%	100.00%	94.44%	53 (4)	183 (10)
	Vif 32→40$_{,36K}$	SKKC**K**GWFY[4]	14/16	N	N/A	N/A	77.77%	44 (8)	146 (4)
	Vif 32→40$_{,36N}$	SKKC**N**GWFY[4] (HLA-B*15:03)	2/16	N	N/A	N/A	66.66%	0	4 (2)
01095-M4	Pol 313→321$_{,320T}$	AIFQSSM**T**K[4]	32/35	Y	100.00%	88.88%	100.00%	559 (20)	795 (12)
	Pol 313→321$_{,320I}$	AIFQSSM**I**K[4] (HLA-A*03:01, A*11:01)	3/35	Y	88.88%	77.77%	88.88%	411 (32)	593 (27)
	Pol 273→290	VPLDKDFRKYTAFTIPSI	35/35	Y	88.88%	94.44%	100.00%	393 (15)	498 (19)
	Gag 397→414$_{,406K,411K}$	REGHIAKNC**K**APRK**K**GCW	8/35	Y	83.33%	77.77%	88.88%	68 (15)	52 (7)
	Gag 397→414$_{,406R,411R}$	REGHIAKNC**R**APRK**R**GCW	5/35	Y	94.44%	77.77%	88.88%	77 (10)	61 (14)
	Gag 397→414$_{,406R,411K}$	REGHIAKNC**R**APRK**K**GCW	19/35	Y	88.88%	83.33%	94.44%	55 (4)	58(7)
	Pol 353→370$_{,355A,359E,366K}$	HR**A**KIE**E**LRQHLL**K**WGFT	11/35	Y	94.44%	88.88%	88.88%	76 (14)	105 (7)
	Pol 353→370$_{,355I,359E,366K}$	HR**I**KIE**E**LRQHLL**K**WGFT	6/35	Y	88.88%	88.88%	88.88%	76 (14)	108 (8)
	Pol 353→370$_{,355I,359E, 366R}$	HR**I**KIE**E**LRQHLL**R**WGFT	2/35	Y	83.33%	94.44%	94.44%	89 (10)	103 (7)
	Pol 353→370$_{,355A,359K,366K}$	HR**A**KIE**K**LRQHLL**K**WGFT	1/35	Y	88.88%	83.33%	83.33%	4 (SEM)	5 (SEM)
	Pol 353→370$_{,355A,359E,366R}$	HR**A**KIE**E**LRQHLL**R**WGFT	15/35	Y	88.88%	94.44%	94.44%	84 (5)	82 (9)

[1] HIV outgrowth assays were performed at limiting dilution in each participant prior to vaccination. Near full-length virus sequences were produced using PacBio sequencing (see Methods). 00749 and 01095 sequences were previously published in (42). Sequences deposited GenBank.[2] HIVconsvX sequences published in (7) [3] Clade B sequence (www.lanl.gov) [4] Each participant was first mapped against overlapping peptides spanning HIV Clade B. The corresponding 18-mer autologous outgrowth reservoir virus peptide/s for all previously defined reactive 18-mers from the initial mapping ELISpot were also synthesized. In addition, optimal CD8 T-cell peptides predicted through LANL's epitope location finder ('ELF') software based on HLA-prediction software (https://www.hiv.lanl.gov/content/sequence/ELF/epitope_analyzer.html), and 18-mer peptides spanning regions where > 40% of the sequences contain non-synonymous mutations in the same position were synthesized. These autologous sequences were then tested in ex vivo ELISpot pre- and post-vaccination to produce the final list of reactive autologous epitopes within each participant. 'Negative' data to variant sequences are also shown.

[5] Empirically confirmed optimal CD8 T-cell epitope (restricting HLA allele).

Supplementary Table 6: Mos-1- and Mos-2-specific CD4+ and CD8+ T cells detected by intracellular cytokine staining (ICS) measured post-vaccination.

Study Arm	Participant	Set at birth	Raace[1]	Ethnicity[2]	Days from vaccination [3]	ICS (background -subtracted frequency)							
						CD4 T cells				CD8 T cells			
						IFN-g +		IFN-g+CD107a+		IFN-g+		IFN-g+CD107a+	
						Mos-1	Mos-2	Mos-1	Mos-2	Mos-1	Mos-2	Mos-1	Mos-2
**M3**	00836	M	AA	NH	7	**0.158%**	**0.174%**	**0.074%**	**0.089%**	*0.021%*	*0.016%*	**0.026%**	**0.017%**
	01237a	M	U	H	56	**0.045%**	**0.128%**	*0.012%*	*0.016%*	**0.160%**	**0.123%**	**0.150%**	**0.093%**
	01249	M	C	NH	14	**0.258%**	**0.252%**	**0.033%**	**0.036%**	**0.024%**	**0.034%**	*0.011%*	*0.007%*
	00937	F	AA	NH	56	**0.106%**	**0.058%**	**0.017%**	*0.009%*	**0.203%**	**0.118%**	**0.209%**	**0.119%**
	00835	M	C	NH	7	**0.104%**	**0.064%**	**0.015%**	**0.015%**	**0.100%**	**0.026%**	**0.098%**	**0.028%**
	01214	M	C	NH	7	**0.086%**	*0.051%*	*0.022%*	*0.016%*	*0.0%*	*0.005%*	*0.006%*	*0.020%*
	01009[1]	F	AA	NH	7	**0.041%**	*0.027%*	**0.007%**	*0.005%*	**0.100%**	**0.100%**	**0.098%**	**0.108%**
**M4**	00749	M	AA	NH	7	*0.043%*	*0.037%*	*0.003%*	*0.004%*	**0.123%**	**0.477%**	**0.065%**	**0.246%**
	01262	M	C	H	56	**0.045%**	**0.047%**	*0.014%*	*0.015%*	*0.023%*	*0.008%*	**0.056%**	**0.036%**
	01264	M	AA	NH	56	**0.088%**	**0.126%**	*0.001%*	*0.000%*	**0.432%**	**0.081%**	**0.278%**	**0.056%**
	01276	M	AA	NH	7	**0.015%**	**0.026%**	*0.006%*	*0.011%*	**0.182%**	**0.217%**	**0.186%**	**0.216%**
	01277[1]	M	AA	NH	14	**0.264%**	**0.298%**	**0.017%**	**0.019%**	**0.369%**	**0.272%**	**0.368%**	**0.268%**
	01095	F	C	NH	14	**0.113%**	**0.085%**	**0.012%**	**0.018%**	**0.622%**	**0.058%**	**0.621%**	**0.061%**
	00115	M	C	NH	7	**0.165%**	**0.139%**	**0.040%**	**0.039%**	*0.0%*	**0.133%**	**0.018%**	**0.127%**
**M3M4**	00631	M	AA	NH	7	**0.068%**	**0.092%**	**0.039%**	**0.050%**	**0.049%**	**0.096%**	**0.050%**	**0.085%**
	00371[1]	M	AA	NH	7	**0.082%**	**0.095%**	*0.0%*	*0.000%*	**0.050%**	**0.424%**	*0.013%*	**0.180%**
	01153[2]	M	C	NH	7	*0.037%*	*0.049%*	*0.004%*	*0.008%*	*0.011%*	*0.005%*	*0.003%*	*0.003%*
	01280	M	C	NH	56	**0.081%**	**0.088%**	**0.030%**	**0.024%**	*0.0%*	*0.0%*	*0.003%*	*0.005%*
	01213	F	AA	NH	14	**0.090%**	**0.086%**	*0.006%*	*0.007%*	*0.008%*	*0.009%*	*0.0%*	**0.009%**
	01295[1]	M	AA	NH	14	*0.021%*	**0.055%**	*0.001%*	*0.000%*	**0.049%**	**0.065%**	**0.058%**	**0.055%**
	01293	M	AA	NH	56	**0.438%**	**0.427%**	*0.013%*	*0.010%*	**0.642%**	**0.378%**	**0.460%**	**0.290%**

[1] Participant expresses one or more of the following HLA I alleles, B*57:01/03, B*58:01, B*81:01 (detailed Supplementary Table 1)
[2] 001153 ICS performed on Day 7 post-vaccination. Average T-cell responses measured by IFN-γ ELISpot at baseline (average two pre-vaccination visits) was Mos-1 105 SFU/10^6 and Mos-2 39 SFU/10^6 and average on Day 7 for Mos-1 was 354 SFU/10^6 and Mos-2 was 221 SFU/10^6
[3] To maximize assay sensitivity, ICS was performed at or near the peak measured HIVconsvX-specific T cell response by IFN-γ ELISpot.
Bold = Mos-1- or Mos-2-specific T-cell response defined by ≥2×mock-stimulated cells and functional gate of Mos-1- or Mos-2-stimulated cells had ≥25 events than corresponding functional gate of mock stimulated cells. Negative measurements indicated in *gray italic.*

Supplementary Table 7: The association of age at enrollment on T-cell response to MVA.HIVconsvX vaccination in PWH on ART[1] estimated from simple linear regression

	Age	Mos-1	Mos-2
Fold-change per 10 person years from baseline to D14 post-vaccination	20	7.30	7.64
	30	5.06	5.32
	40	3.50	3.70
	50	2.43	2.58
	60	1.68	1.79
Fold-change per 10 person years from baseline to D70 post-vaccination	20	5.95	4.61
	30	4.05	3.37
	40	3.50	2.46
	50	1.88	1.80
	60	1.68	1.31

Supplementary Table 8: Age at enrollment and years on ART predict post-vaccination T-cell response following MVA.HIVconsvX vaccination

Antigen	log2FC from baseline to day	Predictors	Slope Estimate[1]	p-value[2]	Multiple R2
Mos-1	7	Age	-0.046	1.74 ×10-2	0.28
	14	Age	-0.053	1.64 ×10-2	0.27
	70	Age	-0.056	6.90 ×10 -3	0.36
	7	Age<br>Years on ART	-0.109<br>0.236	5.03 ×10-4<br>7.63 ×10-3	0.53
	14	Age<br>Years on ART	-0.110<br>0.233	6.24 ×10-4<br>1.15 ×10-2	0.49
	70	Age<br>Years on ART	-0.075<br>0.082	1.79 ×10-2<br>ns	0.39
	7	Age<br>Pre-vax logIPDA	-0.030<br>-0.501	ns<br>ns	0.39
	14	Age<br>Pre-vax logIPDA	-0.034<br>-0.526	ns<br>ns	0.35
	70	Age<br>Pre-vax logIPDA	-0.047<br>-0.381	ns<br>ns	0.42
Mos-2	7	Age	-0.042	2.54 ×10-3	0.41
	14	Age	-0.052	5.52 ×10-3	0.34
	70	Age	-0.045	8.13 ×10-3	0.35
	7	Age<br>Years on ART	-0.028<br>-0.052	ns<br>ns	0.43
	14	Age<br>Years on ART	-0.055 0.011	ns<br>ns	0.34
	70	Age<br>Years on ART	-0.038<br>-0.030	ns<br>ns	0.35
	7	Age<br>Pre-vax logIPDA	-0.040<br>0.035	0.018<br>ns	0.36
	14	Age<br>Pre-vax logIPDA	-0.050<br>0.142	0.024<br>ns	0.32
	70	Age<br>Pre-vax logIPDA	-0.043<br>-0.067	ns<br>ns	0.31

[1] Models are simple or multiple linear regression estimates
[2] ns - non-significant p>0.05
FC=fold change, ART antiviral therapy

Supplementary Table 9: Key Reagents and Resources

REAGENT or RESOURCE	SOURCE	IDENTIFIER
Antibodies – flow cytometry		
CD107a-APC	Biolegend	Cat# 328620, RRID:AB_1279055
CD3-PerCP-Cy5.5	Biolegend	Cat# 300430, RRID:AB_893299
CD4-PE-Cy5	Biolegend	Cat# 317412, RRID:AB_571957
CD8-BV510	Biolegend	Cat# 344732, RRID:AB_2564624
CD14-BV650	Biolegend	Cat# 301836, RRID:AB_2563799
CD16-BV650	Biolegend	Cat# 302042, RRID:AB_2563801
CD19-BV650	Biolegend	Cat# 302238, RRID:AB_2562097
CD56-BV650	Biolegend	Cat# 318344, RRID:AB_2563838
CD45RO-BV605	Biolegend	Cat# 304238, RRID:AB_2562153
IFN-γ-PE	Biolegend	Cat# 502509, RRID:AB_315234
TNFα-PE-Dazzle 594	Biolegend	Cat# 502946, RRID:AB_2564173
MIP-1β-PE-Cy7	BD Biosciences	Cat# 560687, RRID:AB_1727566
Perforin-BV421	Biolegend	Cat# 353307, RRID:AB_11149688
Granzyme B-FITC	Biolegend	Cat# 372206, RRID:AB_2687030
Zombie NIR	Biolegend	Cat# 423106
Antibodies – mass cytometry		
CD45 HI30 111Cd	Standard BioTools	Cat# 3111001B
CCR6 11A9 141Pr	Standard BioTools	Cat# 3141014A
CD19 HB19 142Nd	Standard BioTools	Cat# 3142001B
CD45RA HI100 143Nd	Standard BioTools	Cat# 3143006B
CD4 RPA-T4 145Nd	Standard BioTools	Cat# 3145001B
CCR4 L291H4 149Sm	Standard BioTools	Cat# 3149029A
CD14 M5E2 151Eu	Standard BioTools	Cat# 3151009B
CXCR5 RF8B2 153Eu	Standard BioTools	Cat# 3153020B
PD-1 EH12.2H7 155Gd	Standard BioTools	Cat# 3155009B
CXCR3 G025H7 156Gd	Standard BioTools	Cat# 3156004B
CD27 L128 158Gd	Standard BioTools	Cat# 3158010B
FoxP3 259D/C7 159Tb	Standard BioTools	Cat# 3159028A
CD28 CD28.2 160Gd	Standard BioTools	Cat# 3160003B
Ki-67 B56 162Dy	Standard BioTools	Cat# 3162012B
CD95 DX2 164Dy	Standard BioTools	Cat# 3164008B
CD127 A019D5 165Ho	Standard BioTools	Cat# 3165008B
CCR7 G043H7 167Er	Standard BioTools	Cat# 3167009A
CD8 SK1 168Er	Standard BioTools	Cat# 3168002B
CD25 2A3 169Tm	Standard BioTools	Cat# 3169003B
HLA-DR L243 170Er	Standard BioTools	Cat# 3170013B
CCR5 NP-6G4 171Yb	Standard BioTools	Cat# 3171017A
CD38 HIT2 172Yb	Standard BioTools	Cat# 3172007B
CD56 N901 176Yb	Standard BioTools	Cat# 3172007B
CD16 3G8 209Bi	Standard BioTools	Cat# 3209002B
CD45 HI30 89Y	Standard BioTools	Cat# 3089003B
CD31 WM59 144Nd	Standard BioTools	Cat# 3144023B
CD3 UCHT1	Biolegend	Cat# 300443
CD99 3B2/TA8	Biolegend	Cat# 371302
Bcl-2 100	Biolegend	Cat# 658702
TCRγδ B1	Biolegend	Cat# 331202
CD57 HCD57	Biolegend	Cat# 359602

CCR10 314305	R&D systems	Cat# MAB3478
CD3 UCHT1 AF488	Biolegend	Cat# 300415, RRID: AB_389310
CD4 OKT4 BV650	Biolegend	Cat# 317436, RRID: AB_2563050
CD8 SK1 BV510	Biolegend	Cat# 344732, RRID: AB_2564624
CD14 M5E2 PerCP Cy5.5	Biolegend	Cat# 325622, RRID: AB_893250
CD19 HIB19 PerCP Cy5.5	Biolegend	Cat# 302230, RRID: AB_2073119
CD56 HCD56 PerCP Cy5.5	Biolegend	Cat# 318322, RRID: AB_893389
CD16 3G8 PerCP Cy5.5	Biolegend	Cat# 302028, RRID: AB_893262
Biological samples		
Human PBMCs	Human	Assigned de-identifying code
Buffy Coats	Human	Not applicable
Tissue Culture		
Fetal Calf Serum (ex vivo studies)	Corning	Cat# 35-079-CV
Human AB serum (short term cell lines)	GemCell™	SKU# 100-512-100
IL-2	PeproTech	Cat# 200-02-50UG
RPMI-1640	Gibco	Cat# 61870036
L-Glutamine	VWR	Cat # 97068-088
Sodium Pyruvate	Gibco	Cat# 11360070
Chemicals, peptides, and recombinant proteins		
Phytohemagglutinin	Thermofisher	Cat# 10576015
Cell-ID Intercalator-Ir	Standard BioTools	Cat# 201192A
Cell Stimulation Cocktail (500X)<br>(PMA / Ionomycin)	eBioscience™	00497093
Peptides	Sigma-Aldrich	VC80023
Cell-ID Cisplatin-195Pt	Standard BioTools	Cat# 201195
Critical commercial reagents		
Maxpar X8 Antibody Labeling 147Sm	Standard BioTools	Cat# 201147A
Maxpar X8 Antibody Labeling 152Sm	Standard BioTools	Cat# 201152A
Maxpar X8 Antibody Labeling 173Yb	Standard BioTools	Cat# 201173A
Maxpar X8 Antibody Labeling 175Lu	Standard BioTools	Cat# 201175A
Maxpar X8 Antibody Labeling 166Er	Standard BioTools	Cat# 201166A
Ca^{2+} Mg^{2+} free PBS	Rockland	Cat# MB008
Maxpar Cell Staining Buffer	Standard BioTools	Cat# 201068
Human TruStain FcX	Biolegend	Cat# 422301
FoxP3 Fixation/Permeabilization kit	Thermofisher	Cat# 00-5523-00
Zombie NIR Fixable Viability Kit	Biolegend	Cat# 423106
CD3/28 Human Dynabeads	Gibco™	Cat# 11131D
CD4+ T cell isolation kit	Miltenyi Biotec	Cat# 130-096-533
Deposited data		
Preprocessed mass cytometry data	Goonetilleke Group, UNC	Cytobank Premium, Beckman Coulter
GenBank Sequencing data	Swanstrom /Zhou Groups, UNC.	Gen Bank under PRJNA666896, MT307344-MT308415 and MW054719-MW054856 All data generated (raw)
Software and Algorithms		
Prism v9	Graphpad	https://www.graphpad.com/
FlowJo v10.8.1	BD Biosciences	https://www.flowjo.com/

Python v3.6	Python Software Foundation	https://www.python.org/
Mixcr v2.1.9.6	Bolotin et al. (43, 44)	https://github.com/milaboratory/mixcr/tree/v2.1.9
R v4.2.2	R Core Team	https://cran.r-project.org/
Cytobank v9.3	Cytobank	https://unc.cytobank.org/cytobank
Muscle v5.1	(45)	https://github.com/rcedgar/muscle/releases
Ninja v1.2.2	https://wheelerlab.org/software/ninja/files/NINJA_preprint.pdf	https://github.com/ninja-build/ninja/releases

Supplementary Figure 1: M&M Study Flowchart of participants enrollment

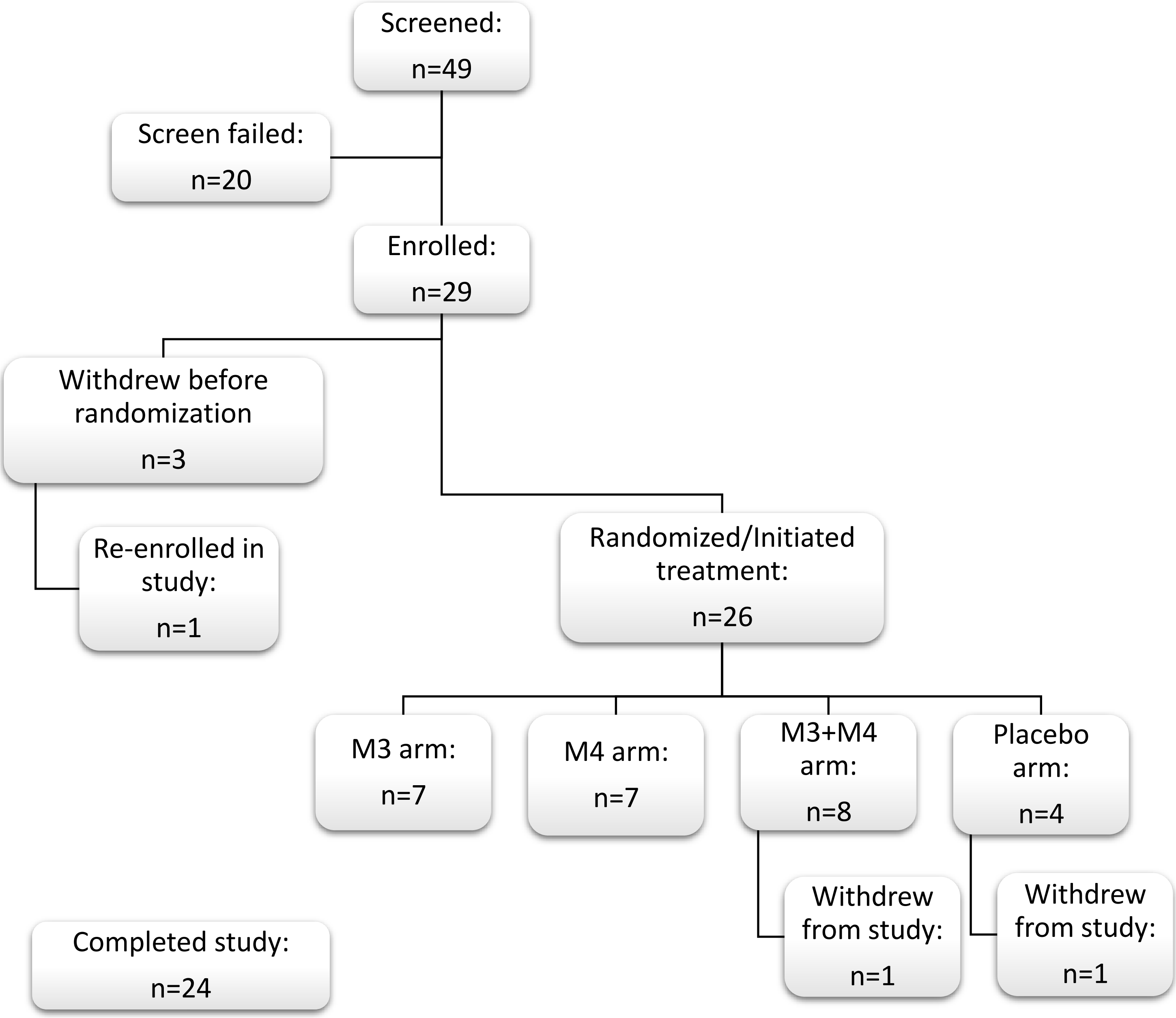

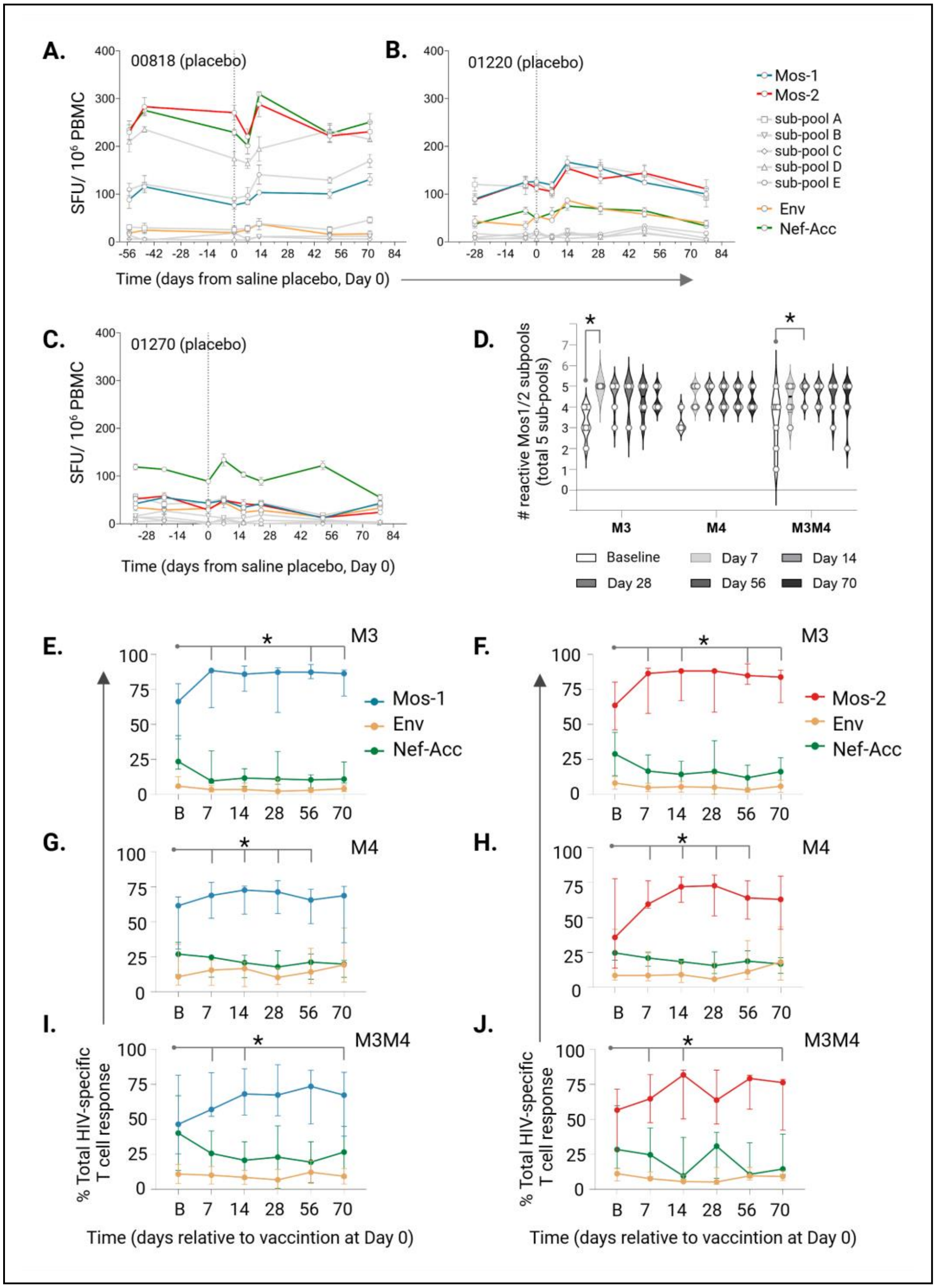

Supplementary Figure 2: **Vaccination with MVA-vectored HIVconsvX immunogens produce significant increases in the frequency of HIV-1-specific T-cells**. (**A-C**) Frequency of HIV-1-specific T-cells (mean +/- SEM) measured by ex vivo IFN-γ ELISpot in participants (**A**) 00818, (**B**) 01220 and (**C**) 01270 receiving saline placebo at day 0. (**D**) Within vaccine arm (n=7/group) changes in T-cell breadth from baseline. Average and 95% CI. (**E-J**) Median and IQR of %Mos-1 (left, E, G, I) and %Mos-2 (right, F, H, J) T-cell frequencies relative to total measured HIV-1-specific T-cell frequency in each vaccine group (n=7/group). %Env and %NEF-ACC also shown. Total frequency = sum of (Mos-1 or 2), Env, NEF and ACC.

Baseline was defined as the average of two pre-vaccination visits, at Day 0 and a previous visit mostly occurring between days -28 to -7. ACC in NEF/ACC peptide pool spans clade B Rev, Tat, Vif, Vpu, Vpr. (D-J) Statistically significant comparisons (*) between baseline (B) and post-vaccination visits are identified with downward ticks, Wilcoxon signed-ranked test. * = $p < 0.05$

Supplementary Figure 3: (**A**) Phylogenetic trees displaying 5' half genome sequences for 00749 (blue), 01905 (green) and 00371 (red). The genetic distance is shown by scale bar (indicated with an asterisk), 1 substitution out of 1000 bp. Trees are midpoint-rooted. (**B**) Group pairwise distance for 5' and 3' genomes.

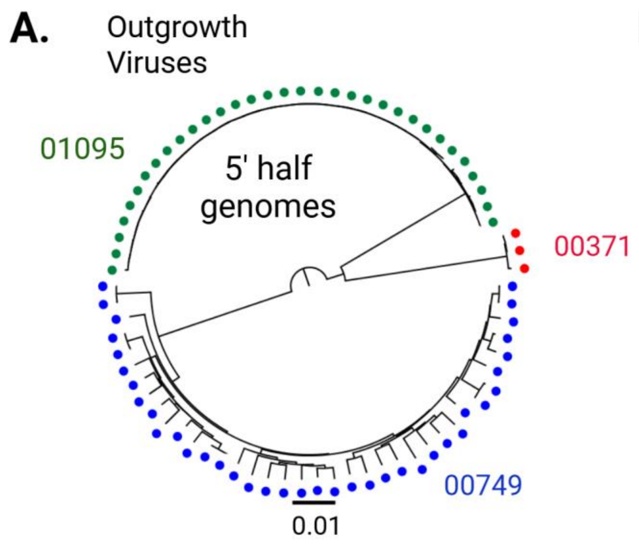

B.

within group pairwise distance

PID	5' half genome	3' half genome
00371	0.00059	0.00099
01095	0.0103	0.0140
00749	0.00041	0.00105

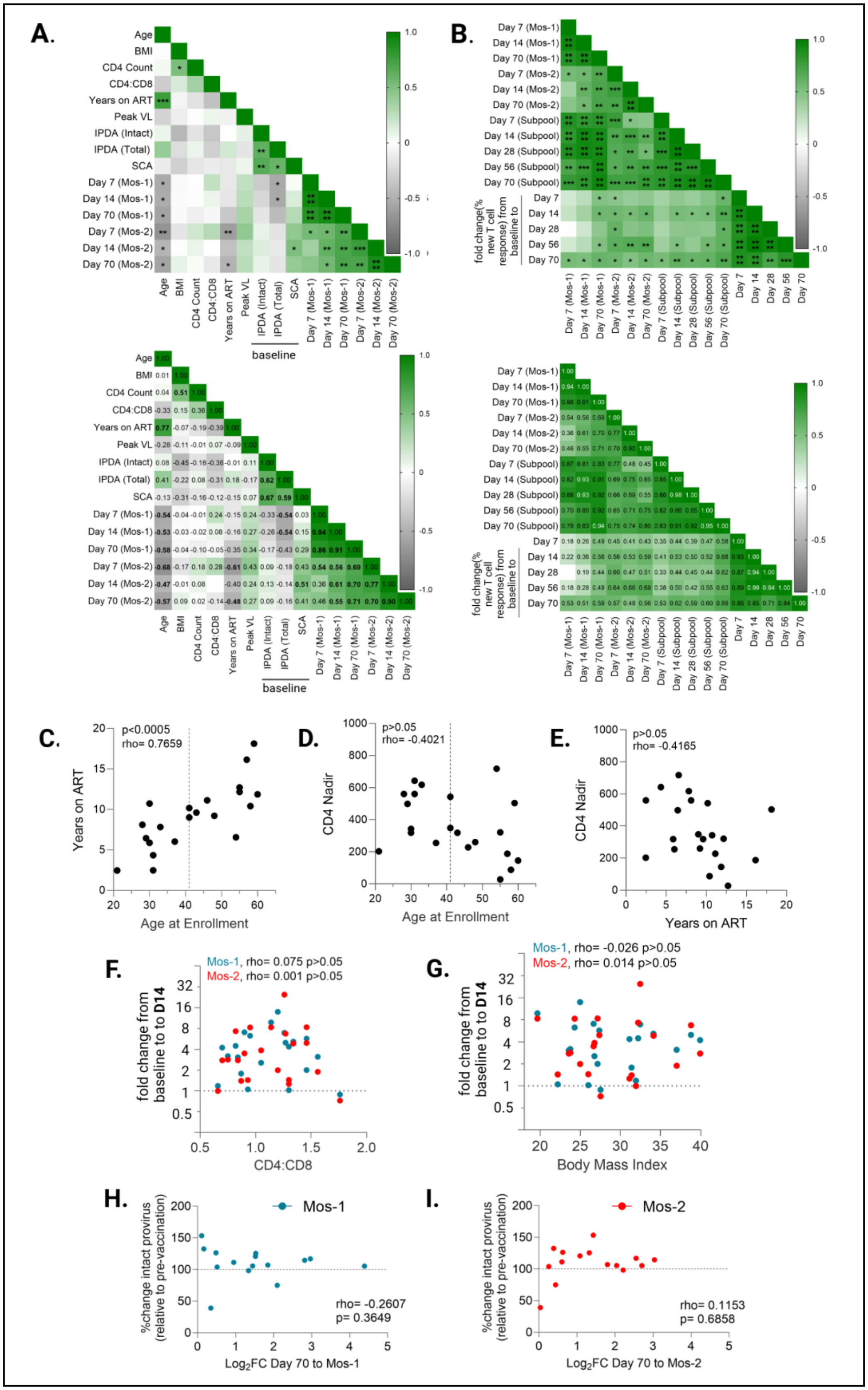

Supplementary Figure 4: **Demographic and clinical data, particularly age, were associated with T-cell response to vaccination with MVA.HIVconsvX vaccines**. (**A-I**) All associations Spearman Rank, two-tailed. (**A**) Correlation matrix between fold-change in T-cell response to Mos-1 and Mos-2 from baseline (average of two pre-vaccination visits) to indicated post-vaccination visit, demographic data and baseline measurements of persistent viremia. Upper panel shows significance levels, lower panel rho values. Age = age at enrolment, BMI = body mass index, SCA = single copy assay, IPDA = integrated proviral DNA assay. CD4 count and CD4:CD8 = values at enrolment (**B**) Correlation matrix between fold-change in T-cell response to Mos-1 and -2, Mos-1/2 sub-pools and %new T cells detected following vaccination across all vaccinees (n=21). Upper panel shows significance levels, lower panel rho values. New T-cells were defined by T-cell response to Mos1/2 sub-pools not detected at baseline timepoints but detected at two or more post-vaccination timepoints. (**C-G**) Associations between selected clinical and or demographic data in vaccinees (n=21). (**H-I**) %Change in intact IPDA® from pre-vaccination (median day -7) to post-vaccination (median day 28) versus log2FC in frequency of Mos-1 (**H**) and Mos-2 (**I**) T-cell frequency for vaccinees (n=17). * = p < 0.05, ** = p < 0.005, *** p < 0.0005, **** p < 0.00005

Supplementary Figure 5: Gating strategy for intracellular cytokine staining. Fluorescence minus one (FMO) controls were used to inform gates for functional markers.

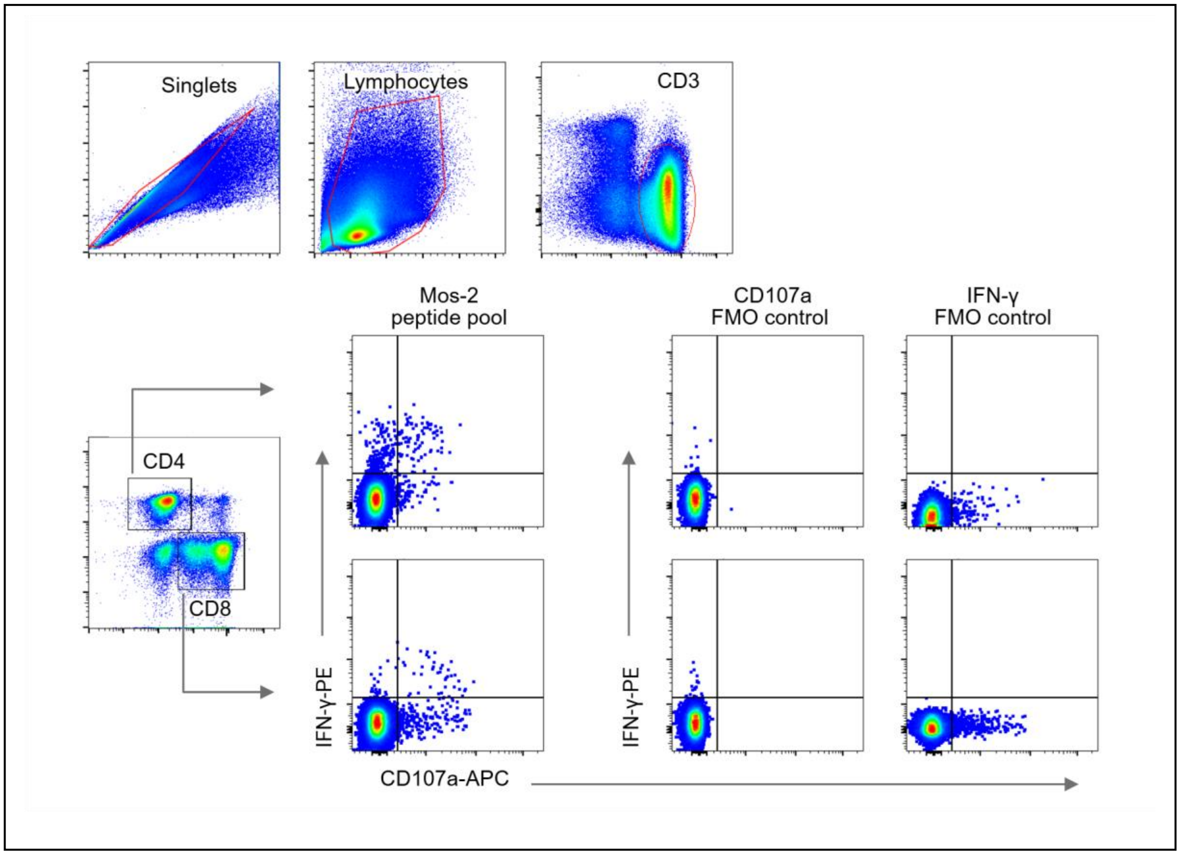

Supplement: Supplemental data [file jci-136-193547-s094.pdf]
